# Platinum–Nickel Nanowires with Improved Hydrogen Evolution Performance in Anion Exchange Membrane-Based Electrolysis

**DOI:** 10.1021/acscatal.0c01568

**Published:** 2020-07-07

**Authors:** Shaun M. Alia, Mai-Anh Ha, Chilan Ngo, Grace C. Anderson, Shraboni Ghoshal, Svitlana Pylypenko

**Affiliations:** † Chemistry and Nanoscience Center, National Renewable Energy Laboratory, 15013 Denver West Parkway, Golden, Colorado 80401, United States; ‡ Computational Science Center, National Renewable Energy Laboratory, 15013 Denver West Parkway, Golden, Colorado 80401, United States; § Department of Chemistry, Colorado School of Mines, 1012 14th Street, Golden, Colorado 80401, United States

**Keywords:** low-temperature electrolysis, anion exchange membrane, electrocatalysis, hydrogen evolution, extended surfaces

## Abstract

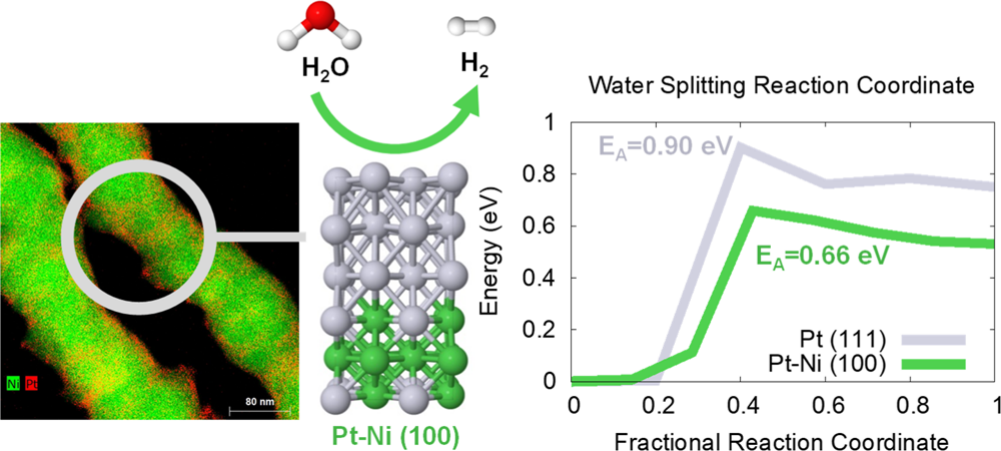

Platinum–nickel (Pt–Ni) nanowires were developed as hydrogen evolving catalysts for anion exchange membrane electrolyzers. Following synthesis by galvanic displacement, the nanowires had Pt surface areas of 90 m^2^ g_Pt_^–1^. The nanowire specific exchange current densities were 2–3 times greater than commercial nanoparticles and may benefit from the extended nanostructure morphology that avoids fringe facets and produces higher quantities of Pt{100}. Hydrogen annealing was used to alloy Pt and Ni zones and compress the Pt lattice. Following annealing, the nanowire activity improved to 4 times greater than the as-synthesized wires and 10 times greater than Pt nanoparticles. Density functional theory calculations were performed to investigate the influence of lattice compression and exposed facet on the water-splitting reaction; it was found that at a lattice of 3.77 Å, the (100) facet of a Pt-skin grown on Ni_3_Pt weakens hydrogen binding and lowers the barrier to water-splitting as compared to pure Pt(100). Moreover, the activation energy of water-splitting on the (100) facet of a Pt-skin grown on Ni_3_Pt is particularly advantageous at 0.66 eV as compared to the considerably higher 0.90 eV required on (111) surfaces of pure Pt or Pt-skin grown on Ni_3_Pt. This favorable effect may be slightly mitigated during further optimization procedures such as acid leaching near-surface Ni, necessary to incorporate the nanowires into electrolyzer membrane electrode assemblies. Exposure to acid resulted in slight dealloying and Pt lattice expansion, which reduced half-cell activity, but exposed Pt surfaces and improved single-cell performance. Membrane electrode assembly performance was kinetically 1–2 orders of magnitude greater than Ni and slightly better than Pt nanoparticles while at one tenth the Pt loading. These electrocatalysts potentially exploit the highly active {100} facets and provide an ultralow Pt group metal option that can enable anion exchange membrane electrolysis, bridging the gap to proton exchange membrane-based systems.

## Introduction

Although hydrogen as a chemical commodity contributes to transportation and agriculture, it currently plays a small role in energy conversion overall. Steam methane reformation is also the primary method for hydrogen production, and electrolysis is a relatively small contributor because of the higher production cost.^1^ The increasing use of low-cost, renewable power sources, however, can allow for both higher use of electrolysis to produce hydrogen and higher use of hydrogen as a chemical intermediate.^2^ Although electrolysis-produced hydrogen costs are currently dominated by the retail price of electricity input, directly pairing electrolyzers with renewable power sources can greatly reduce cost. This pairing increases the significance of capital cost and makes reducing the platinum (Pt) group metal (PGM) loading and improving component performance and durability critical.^1^

Low-temperature electrolysis is typically grouped into three types: alkaline, proton exchange membrane (PEM), and anion exchange membrane (AEM) systems. PEM-based electrolyzers produce relatively high current density but may be somewhat limited by the cost of the PGM content (catalyst layers, transport layer coatings, and separator coatings) and durability losses associated with lower PGM loading and environment.^3^ Systems at high pH (alkaline, AEM) offer a stability benefit for non-PGMs as catalysts and other system components.^4^ Compared to alkaline electrolysis, however, AEM-based electrolyzers can produce current densities similar to PEM electrolyzers and potentially allow for dry hydrogen production with backpressure.^5−11^

Many catalyst development studies in AEM electrolysis focus on oxygen evolution materials due to the slow kinetics; the AEM hydrogen evolution reaction (HER), however, is also critical because hydrogen evolution on Pt is 2 orders of magnitude slower in base than in acid.^12−15^ This has been attributed to changes in hydrogen binding strength, specifically, through either the Volmer–Heyrovsky or Volmer–Tafel mechanisms.^15−19^ The Volmer step is often considered the rate-determining step because of the difficulty of abstracting hydrogen from water for the mechanism in alkaline media;^15,18,19^ other studies attribute interactions between adsorbed H with the OH as an anion or coadsorbed to the surface.^16,20,21^ Thus, understanding mechanistically how optimized catalysts may modify adsorption of key intermediates and tune reaction steps for hydrogen evolution is also of significant interest.

Although non-PGMs are viable, low-PGM catalysts offer performance benefits in lower overpotential and orders of magnitude higher kinetic activity. PGM-based HER materials have generally focused on Pt because of higher activity,^16,22−26^ but ruthenium^27−30^ and palladium^31−33^ (often as bifunctional or hydrogen oxidation electrocatalysts)-based catalysts have also been developed. On occasion, Pt–nickel (Ni) nanostructures have been studied^34−38^ but generally produced mass activities comparable to or lower than commercial Pt baselines.^39,40^

This paper evaluates Pt–Ni nanowires for HER in rotating disk electrode (RDE) half-cells and membrane electrode assembly (MEA) single-cells. Although the primary focus was AEM-based electrolysis, these materials were also developed with the hydrogen oxidation reaction (HOR) in mind for reversible fuel cells; throughout this paper, HOR activity is presented and discussed in a limited capacity.^41−43^ The Pt–Ni nanowires developed in this study build upon previous efforts in oxygen reduction catalysts in PEM-based fuel cells, where thicker Pt-skins grown on Ni_3_Pt stabilized the (100) facet to have comparable stability to (111).^44^ Here, composition and processing conditions were tuned to optimize HER performance and incorporate the nanowires into electrolysis MEAs. Theoretical work assessed the catalytic activity of these Pt-skins grown on Ni_3_Pt by identifying the influence of faceting and lattice compression on the binding of key reaction intermediates and pinpointing mechanistic differences between the different facets for HER. This work leverages ongoing programs, evaluating component performance and durability in low-temperature electrolysis and probing atomic-level questions of stability and activity.^9^

## Methods

### Experimental Section

Pt–Ni nanowires were synthesized by spontaneous galvanic displacement with a Ni nanowire (PlasmaChem GmbH) template and potassium tetrachloroplatinate precursor, using previously developed methods.^45^ The nanowires (40 mg) were horn-sonicated in 80 mL of water for 5 min and added to a 250 mL round-bottom flask. The Ni nanowire dispersion was stirred at 500 rpm with a polytetrafluoroethylene paddle and heated to 90 °C in a mineral oil bath. After reaching the reaction temperature, the platinum precursor was dispersed in 15 mL of water and added dropwise to the flask with a syringe pump at the rate of 1 mL min^–1^. The mass of potassium tetrachloroplatinate precursor was varied to produce different compositions; 8.1 mg of potassium tetrachloroplatinate resulted in Pt–Ni nanowires that were 7.1 wt % Pt. Following the Pt precursor addition, the flask contents were maintained at 90 °C for 2 h, then cooled to room temperature and washed by centrifugation, three times in water and once in isopropanol.

Hydrogen annealing was completed at varying temperatures on the as-synthesized Pt–Ni nanowires, 7.1 wt % Pt. The nanowires were loaded into a 2 inch quartz tube in a Lindberg/Blue M split hinge tubular furnace and held at vacuum overnight to dry. Hydrogen was fed into the tube with a back pressure of 500 Torr, and the temperature was increased at the rate of 10 °C min^–1^ to the annealing temperature, where it was held for 2 h. After annealing, the furnace was cooled without active control; once it reached room temperature, the hydrogen flow was stopped and the samples removed. Acid leaching was completed on the hydrogen annealed (275 °C) nanowires by exposure to 0.05 M nitric acid at room temperature for 2 h.

Pt–Ni composition was determined by inductively coupled plasma mass spectrometry (ICP–MS) with a Thermo Scientific iCAP Q in kinetic energy discrimination mode. Nanowire batches were dissolved in aqua regia and diluted to 200, 20, and 2 ppb, matrix-matched to 1.5% hydrochloric acid and 0.5% nitric acid. The instrument was calibrated to a blank, four internal standards, and three Pt–Ni standards (concentrations of 2, 20, and 200 ppb). The ICP–MS Pt and Ni detection limits (internal detection limits) were less than 1 and 10 ppt, respectively. Three measurements were taken per sample at a 0.15 s dwell time, and the standards were verified following every five samples.

X-ray diffraction (XRD) measurements were taken with a Bruker D8 DISCOVER at 40 kV and 35 mA in the 2θ range 13.5–88°. Powders were pressed onto double-sided carbon tape fixed on a glass slide, and diffraction patterns were taken for 1 h. For nanowires after electrochemical conditioning, the catalyst was removed from the working electrode into 1 mL of water by bath sonication. The resuspended ink was briefly centrifuged, the supernatant partially poured off, and the remaining sample sonicated to concentrate. The sample was then pipetted onto carbon tape and dried, and the diffraction patterns were taken for 2 h. Magnified XRD patterns were presented at 37–45° 2θ, normalized to the area of Pt response (characteristic Pt, 3.92 Å, or compressed). Approximations of the Pt lattice spacing were calculated with Rietveld refinement using Match 3.2.2 (interface) and FullProf 2.05.

High-resolution transmission electron microscopy (TEM), scanning TEM, and X-ray energy-dispersive spectroscopy (EDS) were performed with a FEI Talos S/TEM equipped with a ChemiSTEM detector and operated at 300 kV.

RDE working electrodes were prepared by making inks that consisted of 1 mg of nanowires, 7.6 mL of water, and 2.4 mL of isopropanol. After icing the ink for 5 min, 10 μL of Nafion ionomer (5 wt %, Sigma-Aldrich) was added. The ink was sonicated for 30 s and bath-sonicated for 20 min in ice. Graphitized carbon nanofibers (50 mg) were then added to the ink, which was horn-sonicated for 30 s and bath-sonicated for 20 min prior to pipetting 10 μL of ink onto a glassy carbon electrode, inverted on a modulated speed rotator, and rotated at 100 rpm. After pipetting the ink, the electrode rotation was increased to 700 rpm for 20 min while the electrode dried. The ink meanwhile was sonicated (30 s horn, 20 min bath) in ice, and additional drops of ink were added to the working electrode (50 μL in total) to increase the loading. Following this procedure, the working electrode loading was 25.5 μg_Pt–Ni_ cm_elec_^–2^ or 1.8 μg_Pt_ cm_elec_^–2^ (at 7.1 wt % Pt). Pt supported on high surface area carbon (Pt/HSC, 47 wt % Pt, Tanaka Kikinzoku Kogyo) was used as a PGM baseline. Although ruthenium inclusion can improve Pt activity in HER, Pt/HSC was used as the PGM baseline because it produced comparable or higher half-cell activity and single-cell performance (with NREL Gen 2 membrane/ionomer) than previously evaluated commercial Pt–ruthenium nanoparticles (mass activity/performance basis).^39^ In HOR, however, Pt–ruthenium typically outperforms Pt mass activity in half-cells and Pt performance in single-cells.^39,46^ The Pt/HSC baseline leverages past efforts developing RDE and MEA baselines in AEM-based electrolysis.^39,47^ Pt/HSC-coated electrodes used inks containing 7.6 mg of Pt/HSC, 7.6 mL of water, 2.4 mL of isopropanol, and 40 μL of Nafion ionomer. After the ink and electrodes were prepared by previously published methods, 10 μL of ink was pipetted onto glassy carbon electrodes and dried at 700 rpm, resulting in a Pt loading of 17.8 μg_Pt_ cm_elec_^–2^.^48^ The higher Pt loading for Pt/HSC (17.8 μg_Pt_ cm_elec_^–2^) was necessary to reach a diffusion limited current in HOR; conversely, the lower Pt loading for the Pt–Ni nanowires was necessary to avoid the Nernstian diffusion limited overpotential and ensure kinetics was evaluated in half-cell testing. Ni nanoparticles (Alfa Aesar, 45505) were used as the non-PGM baseline. Ni-coated electrodes used inks containing 3.49 mg, 7.6 mL of water, 2.4 mL of isopropanol, and 40 μL of Nafion ionomer. After the ink and electrodes were prepared by previously published methods, 10 μL of ink was pipetted onto glassy carbon electrodes and dried at 700 rpm, resulting in a Ni loading of 17.8 μg_Ni_ cm_elec_^–2^.^39^

RDE testing was completed with a PGSTAT302N (Metrohm Autolab B.V.) potentiostat. Pt catalysts (Pt/HSC, Pt–Ni nanowires) were electrochemically conditioned in 0.1 M perchloric acid in a glass cell with a gold counter electrode and a reversible hydrogen electrode (RHE) filled with the electrolyte and connected to the main cell with a Luggin capillary. Pt catalysts were cycled (Pt/HSC 50 cycles, Pt–Ni 100 cycles) in perchloric acid in the potential range 0.025–1.4 V *versus* RHE at 2500 rpm and 500 mV s^–1^. The Pt catalysts were then rinsed and conditioned in 0.1 M sodium hydroxide electrolyte (TraceSELECT, Honeywell Research Chemicals, 01968), 20 cycles −0.1 to 1 V *versus* RHE at 2500 rpm and 500 mV s^–1^ in a polytetrafluoroethylene cell with a gold counter electrode and a mercury/mercurous oxide reference electrode. Ni nanoparticles were conditioned in base (0.1 M sodium hydroxide) and in the potential range −0.3 to 0.1 V *versus* RHE (20 cycles at 2500 rpm and 500 mV s^–1^) to avoid dissolution (in perchloric acid) and oxide growth (at higher potential range). Following conditioning, polarization curves were taken cathodically at 10 mV s^–1^ and 2500 rpm in the range −0.1 to 1 V (Pt) and −0.3 to 0.1 (Ni) *versus* RHE. HER–HOR polarization curves were corrected for internal resistance (22–25 Ω) with a current interrupter at 0.4 V *versus* RHE and fit to the Butler–Volmer (Pt) and Tafel (Ni) equations. The diffusion limited current (2.7 mA cm_elec_^–2^) was lower than at sea level because of the local elevation (5674 ft) and partial pressure (83.2 kPa), and the calculated exchange current densities were corrected for the hydrogen partial pressure at a reaction order of 0.6.

Electrochemical surface area (ECA) measurements were completed during cyclic voltammograms in a 0.1 M sodium hydroxide electrolyte and determined from the charge associated with hydroxide desorption (Ni) and the oxidation of an adsorbed carbon monoxide monolayer (Pt). Carbon monoxide was adsorbed on Pt surfaces by holding the electrode at 0.1 V *versus* RHE in a carbon monoxide saturated electrolyte for 10 min, followed by 10 min of purging nitrogen to remove excess carbon monoxide. Cyclic voltammograms were taken immediately thereafter, with the carbon monoxide monolayer oxidized/desorbed in the first cycle and complete removal ensured in subsequent cycles. Pt{100} facets were quantified by germanium adsorption. A solution containing 0.01 M germanium(IV) oxide and 1 M sodium hydroxide was pipetted to cover the electrode surface. The electrode was then placed in a three-electrode cell containing 0.5 M sulfuric acid, while being held at 0.1 V *versus* RHE, and immediately cycled in the potential range 0.025–0.6 V *versus* RHE at 50 mV s^–1^. The charge associated with germanium adsorption was normalized to the Pt{100} surface area by 0.56 factor. Pt{111} facets were quantified by tellurium adsorption. Pt-coated electrodes were submerged in a solution containing 10^–4^ M tellurium dioxide and 0.5 M sulfuric acid. After rinsing with water, the electrodes were cycled in the potential range 0.025–0.9 V *versus* RHE at 50 mV s^–1^ in 0.5 M sulfuric acid.

RDE durability testing was completed by cycling in the potential range −0.2 to 0.2 V *versus* RHE, 30,000 cycles at 500 mV s^–1^ in 0.1 M sodium hydroxide electrolyte. This range was used to cover the anticipated operating potentials in both electrolyzer and fuel cell modes (reversible fuel cells). This range did not cross Pt redox and was not expected to accelerate Pt loss but was used to assess the impact of Ni oxide growth on catalysis.

Single-cell MEAs consisted of a perfluorinated AEM and ionomer (NREL Gen 2) and were used because of availability and performance/thicknesses similar to commercial suppliers. Catalyst layers (5 cm^2^) were prepared by spraying inks onto carbon (Toray) porous transport layers (PTLs). Anode catalyst layers consisted of cobalt (Co) nanoparticles (Alfa Aesar) sprayed to a loading of 0.4 mg_Co_ cm^–2^ and an ionomer (NREL Gen 2) to catalyst ratio of 0.22:1. Cobalt anodes were used because they were previously baselined, commercially available, and produced reasonable MEA performance without the use of PGMs.^47^ Cathode catalyst layers were sprayed with metal-based (Pt, Ni) loadings of 0.1 (Pt/HSC, Pt–Ni nanowires) and 0.2 (Ni nanoparticles) mg cm^–2^. Pt/HSC included a carbon support; Pt–Ni nanowires and Ni nanoparticles were sprayed with Ketjenblack in a 1:1 ratio (carbon/metal) to avoid the higher resistances (contact, interfacial) found at low catalyst loading. All cathodes were sprayed with an ionomer (NREL Gen 2) to catalyst (metal and support) ratio of 0.22:1 (ionomer to carbon ratio of 0.44:1). MEAs were assembled with a perfluorinated AEM (NREL Gen 2, ion exchanged to hydroxide form), single-serpentine Ni flow fields, and aluminum end plates (Fuel Cell Technologies, Inc., isolated from the electrolyte).

MEAs were tested at 80 °C in a 1 M potassium hydroxide supporting electrolyte at a flow rate of 0.1 L min^–1^ on the anode and cathode.^47^ Cells were conditioned by a 2 h hold at 2 V, and potentiostatic polarization curves were taken cathodically and then anodically at a 5 min step duration. Cyclic voltammograms were completed in the potential range of 0.025–1.3 V, and impedance was taken in the range of 1–100,000 Hz for each potential used in the polarization curve.

### Theoretical Section

As was previously done on Pt–Ni alloys with a Pt-skin,^44^ all plane wave density functional theory calculations were performed with the Vienna Ab initio Simulation Package^49−52^ using the most updated projector augmented wave pseudopotentials;^53,54^ spin-unrestricted calculations employed the Perdew–Burke–Ernzerhof^55^ functional. The basis set was expanded to a kinetic energy cutoff of 520 eV with stringent convergence criteria of 10^–6^ eV (10^–5^) implemented on electronic (geometric) relaxations. Electronic occupations utilized Gaussian smearing. We focused on Pt–Ni alloys with a thicker Pt-skin of three layers, particularly focusing on a Ni_3_Pt subsurface and an appropriate range of lattice constants noted in both our experimental XRD and other studies: 3.62 (Ni_3_Pt alloy), 3.77 (intermediate between Ni_3_Pt and Pt), and 3.92 (pure Pt) Å.^44,56−59^ The alloy sublayer in contact with the Pt-skin and the Pt-skin were allowed to translate across all three dimensions; all other layers of the alloy were held fixed with respect to the bulk. The alloy sublayer may expose a Ni surface or a Pt–Ni surface on the (100) and (110) facets of Ni_3_Pt, but we previously established that the Pt–Ni layer results in greater stability of the Pt-skins.^44^ Appropriate *k*-point meshes were selected for the size of surfaces: the Pt-skins on Ni_3_Pt utilized (4 × 4 × 1) for (100), (2 × 3 × 1) for (110), and (2 × 2 × 1) for (111); the pure Pt surfaces grown from a unit cell to be a (2 × 2 × 2) surface utilized *k*-point meshes of (4 × 4 × 1). (111) surfaces for both pure Pt and Pt-skins on a Ni_3_Pt surface were hexagonal cells, requiring the *k*-point mesh to be centered at Γ. In the global minimum search for key reaction intermediates (H_2_O, H, OH), ∼120 initial geometries were sampled; this was applied to each Pt and Pt–Ni surface, resulting in ∼1440 calculations in order to determine the lowest energy structures of adsorbed H_2_O, H, and OH. Atomic, bridging, and hollow sites were sampled; adsorbates were also rotated every 45° with H_2_O and OH oriented in different configurations. These global minima are visualized in the Supporting Information (Figures S6–S9).

Adsorption energies were calculated under the convention of *E*_ads_ = *E*_surf+ads_ – *E*_surf_ – *E*_gas,ads_, where *E*_surf+ads_ refers to the total energy of the surface with the adsorbate, *E*_surf_ refers to the total energy of the clean surface, and *E*_gas,ads_ refers to the total energy of the gas phase adsorbate. The reaction profile for the Volmer step assumes in the absence of pressure–volume changes to the system (theoretically, our calculations were at 0 K, in vacuum): H_2_O_ads_ → H_ads_ + OH; H_2_O_ads_ is the total energy of the surface with a single water adsorbed, H_ads_ is the total energy of the surface with a single hydrogen adsorbed, and OH is the total energy of the gas phase OH species. Climbing image nudged elastic band (cNEB) calculations were performed to evaluate the minimum energy pathway to water-splitting on surfaces of interest.^60,61^ In order to get results in a reasonable time frame, four images were propagated between the initial and final states on the larger (111) surface, whereas six images were utilized for the smaller (100) surface.

## Results and Discussion

The Results and Discussion section of this paper has been divided into three sections based on experimental aims and the material set evaluated. The “As-Synthesized Pt–Ni Nanowires” subsection focuses on nanowire synthesis with spontaneous galvanic displacement and establishing a start point for post-synthesis optimization. The “Hydrogen Annealing” subsection focuses on improving HER performance through an alloying effect and the underlying causes for activity improvements. Specifically, experimental characterization of exposed facets and lattice changes was then paired with atomic and electronic level calculations of key adsorbates and reaction steps to water-splitting. The “Material Optimization and MEA Testing” subsection focuses on the modification of Pt–Ni nanowires and their incorporation into MEAs.

### As-Synthesized Pt–Ni Nanowires

Spontaneous galvanic displacement was used to synthesize Pt–Ni nanowires with variable composition. Tuning the amount of Pt precursor produced nanowires with Pt contents less than or equal to 17 wt %; for higher Pt compositions, acid was added to the synthesis flask to remove near-surface Ni oxides and allow for further Pt deposition. Galvanic displacement resulted in morphologies (nanowires and nanotubes) and dimensions similar to the as-received Ni nanowire template, approximately 100–250 nm in diameter and 50–200 μm in length (Figures S1 and S2). Although galvanic displacement did not necessarily produce complete or uniform Pt coatings, the coatings appeared approximate at or near the surface and tended to segregate into zones separate from the Ni lattice.^45^

Following synthesis, the Pt–Ni nanowires were studied for HER−HOR activity in RDE half-cells. Several choices in testing and evaluation affected the observed performances. Pt-based catalysts were electrochemically conditioned in an acidic electrolyte (0.1 M perchloric acid, 50−100 cycles 0.025–1 V) to remove contaminants and expose Pt sites. Following conditioning in acid, the catalysts were rinsed in water and conditioned in base (0.1 M sodium hydroxide) prior to activity and surface area determinations. Prior to use, a polycrystalline Pt electrode was used to remove contaminants in the alkaline electrolyte (0.1 M sodium hydroxide) by electroplating, previously found to result in similar iron contaminant levels and baseline activities as *ex situ* chemical approaches.^39,47^ Pt HER−HOR activity was evaluated during linear sweep voltammograms in the potential range of −0.1 to 1.0 V cathodically to avoid hydrogen bubble formation partially covering the electrode and reducing the performance. Current responses in the kinetic region were fit to the Butler–Volmer equation to determine exchange current densities. Although many publications compare relative performances based on overpotential, exchange current densities may be more appropriate for RDE testing for reactions with fast kinetics, and overpotentials may exaggerate the relative importance of kinetics (*vs* Ohmic, transport loss) at moderate current densities in MEAs. The anodic charge-transfer coefficients of the Pt–Ni nanowires were approximately 0.5 (α_a_ = 0.46–0.53), which were expected for Pt-based catalysts and indicative of comparable activity in HER and HOR. Occasionally, activities deviated from the Butler–Volmer fitting at HER current densities greater than 35 mA cm^–2^ because of hydrogen bubble formation (transport) and at low HOR overpotential because of hydrogen diffusion limitations to the working electrode (Nernstian diffusion limited overpotential). For the Ni nanoparticle baseline, HOR activity was not observed, and an exchange current density was derived from the Tafel equation (Figure S3).

**Figure 1:**
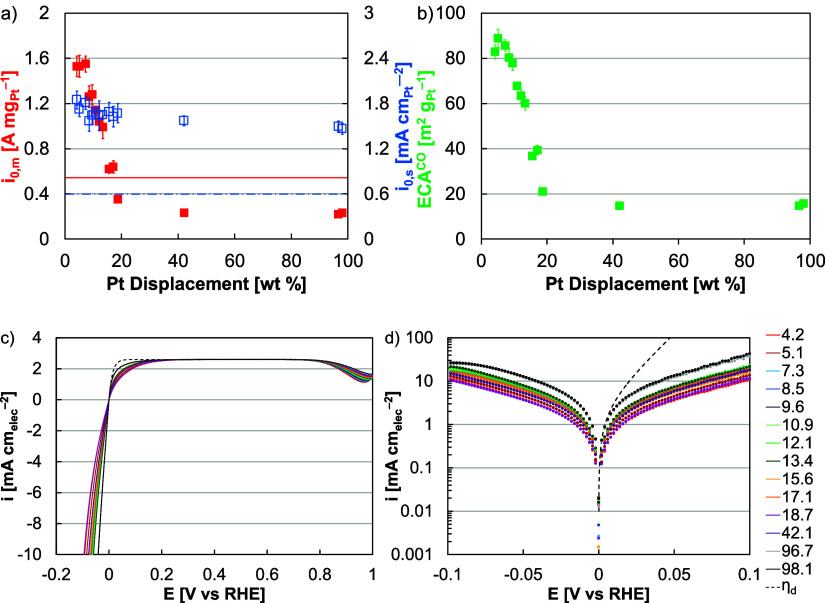
(a) HER–HOR mass (red) and site-specific (blue) exchange current densities of as-synthesized Pt–Ni nanowires in RDE half-cells as a function of Pt content (*x*-axis). The mass (solid red) and site-specific (dashed blue) activities of Pt/HSC are provided as horizontal lines. (b) Pt ECAs (green) of as-synthesized Pt–Ni nanowires as a function of Pt content (*x*-axis). (c) Linear sweep voltammograms and (d) Butler–Volmer plots of as-synthesized Pt–Ni nanowires with the Nernstian diffusion limited overpotential (η_d_, dashed line).

Modifying the Pt content produced trends in HER–HOR activity. First, the as-synthesized nanowires generally produced specific activities 2–3 times greater than carbon-supported Pt (Pt/HSC) in RDE half-cells (Figure 1a). The higher specific activity may have been due to the extended surface avoiding less active sites and fringe facets.^62^ The specific activity of Pt–Ni was generally constant regardless of the composition. At lower levels of displacement, however, a slight increase in specific activity was observed, which may have been due to localized alloying (increased proximity of Pt and Ni zones), modifying Pt–H binding.^25^ On a larger scale (bulk material), however, the Pt lattice was characteristic, and the Pt and Ni zones appeared relatively segregated at low Pt content. The presence of surface Ni may have also contributed to HER–HOR activity by increased oxophilicity or through Ni contributing to HER activity itself.^63^ The majority of surface Ni, however, was removed during electrochemical conditioning in an acidic electrolyte (0.1 M perchloric acid), necessary to expose Pt sites and improve activity (Figure S4). This finding, higher specific activity at lower Pt content, was a deviation from Ni nanowire-templated Pt oxygen reduction catalysts, where increased amounts of near-surface Ni inhibited activity.^45^

Second, the Pt ECA increased at lower displacement levels from 15.7 m^2^ g_Pt_^–1^ at 98.1 wt % Pt to 88.8 m^2^ g_Pt_^–1^ at 5.1 wt % Pt (Figure 1b). Higher Pt ECA at lower Pt content was likely due to the deposition of thinner layers on the nanowire surface improving Pt utilization. A slight drop in ECA was observed at lower Pt compositions, indicating that a minimum Pt content may be necessary to produce an approximate coating and achieve higher surface areas. Because lower Pt content resulted in higher Pt ECAs and comparable specific activities, the HER mass activity generally increased at lower Pt composition (Figure 1a,c,d). The as-synthesized wires produced a maximum mass exchange current density of 1.55 A mg_Pt_^–1^ at 7.1 wt % Pt in RDE half-cells, more than 6 times greater than the fully displaced nanowires (6.7 times, 98.1 wt % Pt) and more than 2 times greater than Pt/HSC (2.8 times, Figure 1a).

### Hydrogen Annealing

**Figure 2:**
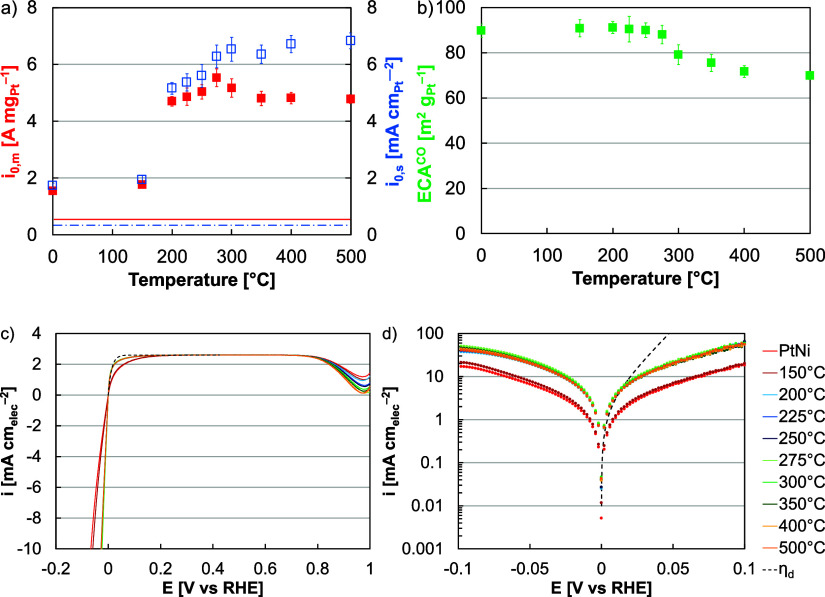
(a) HER–HOR mass (red) and site-specific (blue) exchange current densities of hydrogen annealed Pt–Ni nanowires in RDE half-cells as a function of annealing temperature (*x*-axis). The mass (solid red) and site-specific (dashed blue) activities of Pt/HSC are provided as horizontal lines. (b) Pt ECAs (green) of hydrogen annealed Pt–Ni nanowires as a function of annealing temperature (*x*-axis). (c) Linear sweep voltammograms and (d) Butler–Volmer plots of as-synthesized Pt–Ni nanowires with the Nernstian diffusion limited overpotential (η_d_, dashed line).

**Figure 3:**
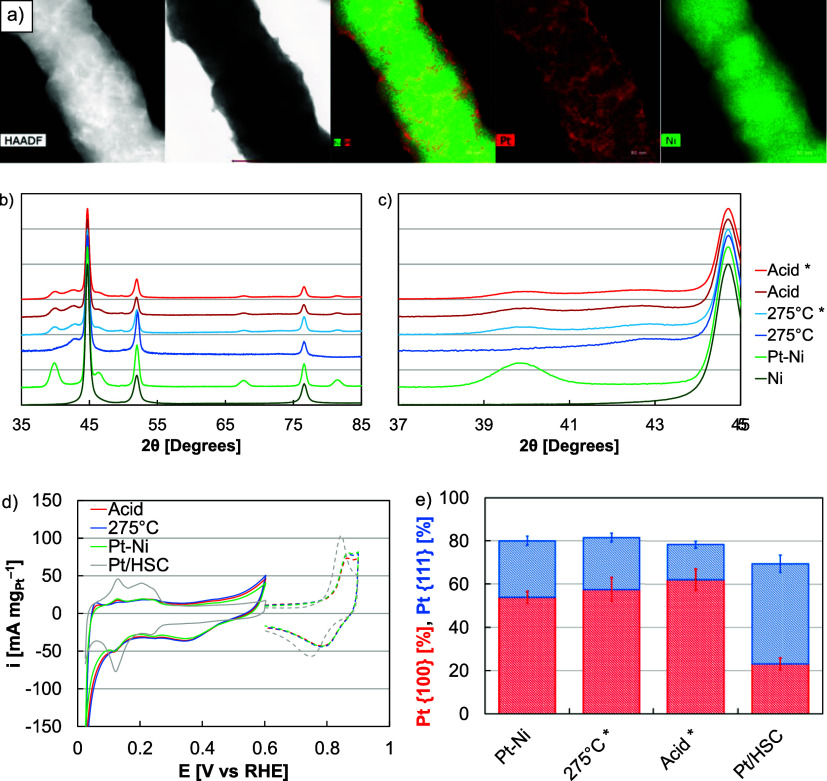
(a) Microscopy of Pt–Ni nanowires hydrogen annealed to 275 °C, including dark-field imaging, bright-field imaging, and EDS of Pt (red) and Ni (green). (b,c) XRD patterns of Ni nanowires (Ni) and Pt–Ni nanowires, as-synthesized (Pt–Ni), hydrogen annealed to 275 °C *ex situ* (275 °C) and following electrochemical conditioning (275 °C *), and acid leached *ex situ* (Acid) and following electrochemical conditioning (Acid *). (d) Cyclic voltammograms of Pt–Ni nanowires (as-synthesized, hydrogen annealed, and acid treated) and Pt/HSC in a germanium (solid) and tellurium (dashed) containing electrolyte. (e) Approximation of exposed Pt facets for Pt–Ni nanowires (as-synthesized, hydrogen annealed, and acid treated) and Pt/HSC based on germanium and tellurium data.

The as-synthesized nanowires with a composition of 7.1 wt % Pt were used as a starting point for post-synthesis optimization because of the high Pt ECA. Hydrogen annealing was then applied to compress the Pt lattice and improve site-specific activity. Higher annealing temperature generally improved HER–HOR activity in half-cell testing, and a large specific activity increase was observed between 150 and 200 °C (Figure 2a). This activity increase was likely due to an alloying effect, with Pt lattice compression modifying the hydrogen binding energy and weakening the Pt–H chemisorption.^16,25^ While increasing amounts of near-surface Ni may have contributed to HER itself or by providing oxophilic sites in close proximity to Pt, the majority of surface Ni was removed during electrochemical conditioning.^63^ At higher annealing temperatures, the Pt–Ni specific activity plateaued. In contrast, the ECA dropped, potentially due to surface segregation and Pt aggregating at higher annealing temperature (Figures 3a and 2b). In terms of mass activity, the Pt–Ni nanowires produced a peak exchange current density of 5.5 A mg_Pt_^–1^ following hydrogen annealing at 275 °C (Figure 2a,c,d). This optimum corresponded to a significantly improved specific activity while retaining a relatively high ECA and was more than 3 times greater than the as-synthesized Pt–Ni nanowires (3.6 times) and 10 times greater than Pt/HSC (10.1 times, Figure 2a).

Analysis of XRD patterns revealed that hydrogen annealing significantly altered the Pt lattice, from relatively segregated (Pt–Ni as-synthesized, clear Pt peak corresponding to 3.92 Å) to heavily compressed at 275 °C, with an average lattice spacing 3.72 Å (Figure 3b,c). In addition to peak shifts that indicated Pt lattice compression, peak broadening was also found (Figure 3c). While broadening can be due to changes in the crystallite size or defects, the asymmetric nature of the broadening suggested that the Pt lattice compression may not be uniform and that hydrogen annealing produced a range of Pt lattices. Electrochemical conditioning in acid was also necessary to expose Pt sites and achieve high activity. This process resulted in the re-emergence of a characteristic Pt peak, indicating a degree of Ni dissolution and Pt expansion (Figure 3b,c). The XRD pattern, however, appeared to indicate that Pt lattice compression was still prevalent and likely improved the HER–HOR activity. Differences in exposed lattices may also have affected the performance (Figures 3d,e and S5). Although hydrogen annealing to 275 °C resulted in slightly more Pt{100} and slightly less Pt{111}, the differences were less than 5%. These results indicated that some heterogeneity in terms of lattice spacing (characteristically Pt to heavily compressed) and facet (although {100} dominant) existed, which may have resulted in a range of chemisorption strengths and activities at individual Pt sites.^16,25,62^

As noted in previous experimental studies, HOR–HER activity suffers a drop moving from acidic to alkaline media.^13,15^ Changes to hydrogen adsorption in particular would significantly alter the mechanism for HER, such as in the Volmer–Heyrovsky or Volmer–Tafel mechanisms^15−19^
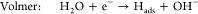

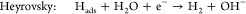

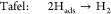


In alkaline media, hydrogen evolution may rely upon the Volmer step, a potentially, energetically uphill reaction requiring water to split to supply adsorbed H (H_ads_);^15,18,19^ moreover, the adsorbed H may have to compete with OH species for active sites in order to form H_2_.^16,20,21^ Detailed theoretical studies for the mechanism in acidic media suggest that the Heyrovsky or Tafel steps may be the rate-determining steps to hydrogen evolution; an experimental study on various Pt facets also agreed with this.^17,64^ For our theoretical study of the mechanism in alkaline media, we investigated changes in the binding strength of key adsorbates such as water (H_2_O_ads_), hydrogen (H_ads_), and hydroxide (OH_ads_) and changes to the reaction energetics and mechanistic barriers at the Volmer step. The visualization of all global minimum structures of adsorbed H, OH, and H_2_O on Pt and Pt–Ni surfaces may be found in Supporting Information, Figures S6–S9 and Tables S1 and S2. We note that we focus on the hydroxide species covalently adsorbed as a hydroxy group to a metal site rather than as a charged anion. Similarly, due to the nontrivial nature of modeling the redox reaction, our reaction profile for the Volmer step is approximated to be H_2_O_ads_ → H_ads_ + OH (assuming e^–^ → OH^–^ is equivalent to OH). For the other possible steps such as the Heyrovsky and Tafel steps, weakened hydrogen binding would most likely aid in the formation and desorption of H_2_ (Supporting Information, Table S1).

**Figure 4:**
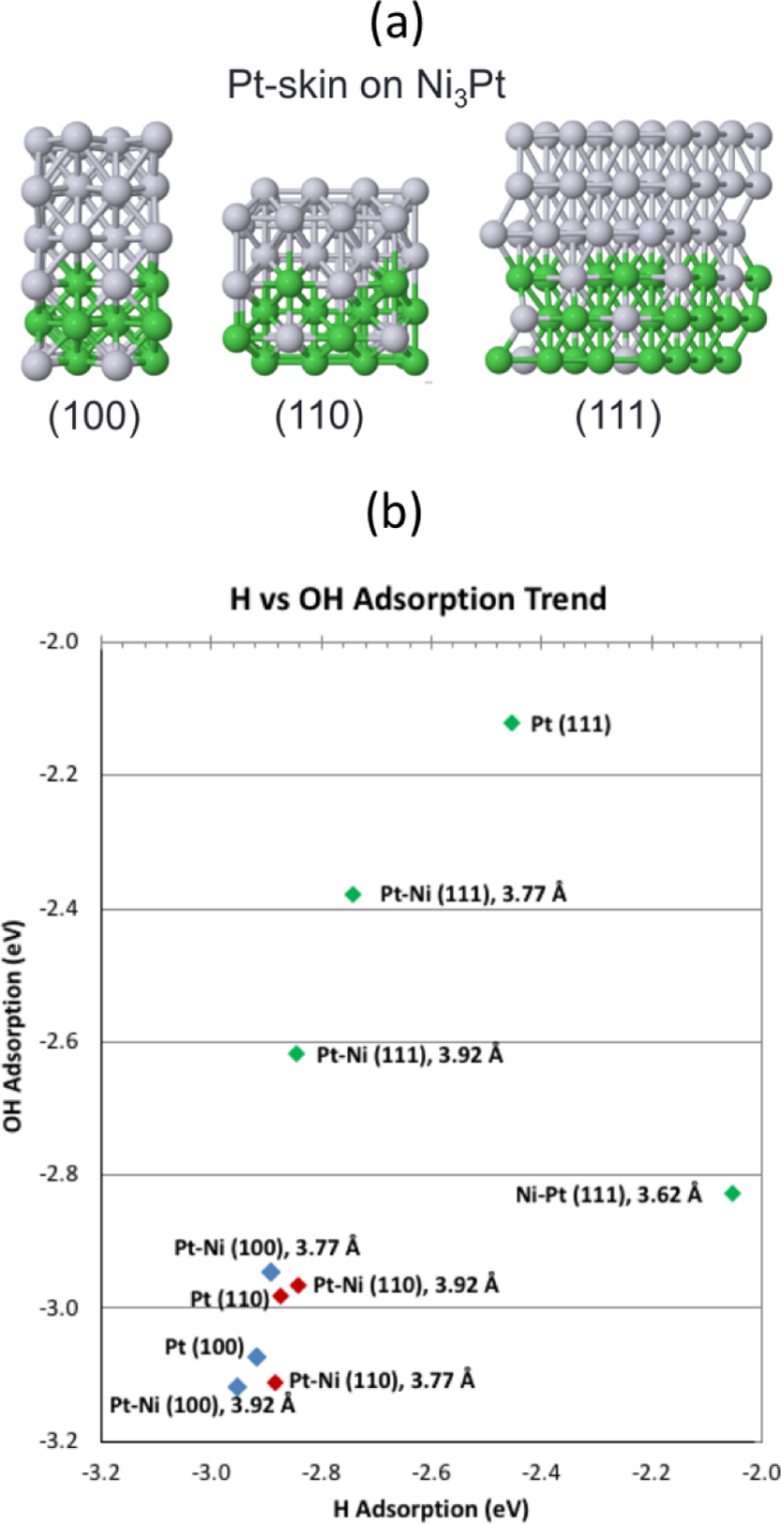
(a) Exposed facets of a thick Pt-skin (three layers of Pt) on the Ni_3_Pt subsurface. Pt atoms are in gray and Ni atoms are in green. (b) Adsorption strength of OH *vs* the adsorption strength of H on pure Pt and the Pt-skins grown on the Ni_3_Pt subsurface. Because of the space constraints on the graph, “Pt–Ni” refers to the Ni_3_Pt subsurface with a Pt-skin, whereas Pt refers to the pure Pt surface. Data point markers are categorized by the facets: green indicates the (111) facet; blue—(100); and red—(110).

Pt–Ni nanowires and baseline Pt catalysts exposed a range of facets, with Pt–Ni exposing a majority of the {100} family and Pt/HSC exposing a majority of the {111} family (Figure 3e). Moreover, the Pt–Ni nanowires display a range of lattice constants (3.60–3.92 Å, Figure 3b). Therefore, Pt–Ni surfaces were modeled with lattices of 3.62, 3.77, and 3.92 Å and facets of (100), (110), and (111) in order to observe changes in the binding strength of key adsorbates because the Pt–Ni nanowires and baseline Pt catalysts exposed some percentage of these facets. It is well known that compression of the lattice may weaken adsorption strength and that different facets can also modify the adsorption strength.^65,66^ However, most of these studies observed this on either pure surfaces (or surfaces with only a single layer of a Pt-skin) or with minor compression, <3%, whereas our surfaces consider a more realistic Pt-skin of three layers (Figure 4a) and compression of up to 7.7% as compared to pure Pt’s lattice of 3.92 Å. On the synthesized Pt–Ni nanowires, there exists a range of composition and heterogeneity of Pt-skin thickness on the Ni core; theoretical calculations focused on a Pt-skin of three layers as a reasonable approximation to both capture thicker Pt-skin and retain the electronic effects of a subsurface Pt–Ni alloy. Subsequent adsorbate calculations required sampling initial geometries >1400 in order to find the global minimum structures of adsorbed H_2_O, H, and OH. For brevity in the plots and in our results and discussion, we will refer to the three layers of Pt-skin grown on Ni_3_Pt as “Pt–Ni.”

Ideally, the alloying effect of Ni to Pt would weaken the binding of not only hydrogen but also the OH species, which may potentially obstruct active metal sites. Tempering the high oxophilicity of Pt sites would aid in the adsorption of H and the formation and desorption of H_2_. In Figure 4b, we plot the adsorption energies of OH *versus* H. On the (100) facet of Pt–Ni, the binding strengths of H and OH are weakened the most at a lattice of 3.77 Å. In contrast, on the (110) facet of Pt–Ni, H and OH binding are weakened the most at a lattice of 3.92 Å, and on the (111) facet of Pt–Ni, H binding is weakened the most at 3.62 Å and OH binding is the weakened the most at 3.77 Å. We note that when the crystalline lattice of the system is compressed to 3.62 Å, some distortion of the surface occurs, and this distortion results in a less stable surface that can favor stronger adsorption of intermediates (Supporting Information, Table S1). Moreover, Pt–Ni surfaces may also display different bonding motifs than pure Pt because of either the compression of the lattice to 3.62 and 3.77 Å or the subsurface effect of Ni_3_Pt (Supporting Information, Figures S6–S9 and Table S2). This can have implications on the mechanism of water-splitting and H_2_ formation. Surprisingly, the Pt–Ni (111) surface with a lattice of 3.92 Å can spontaneously split OH; this is also the global minimum structure when adsorbing OH. Potentially, H_2_ formation may occur on this surface *via* adsorbed H from both water-splitting and OH-splitting.

In addition to moderating the binding strength of key intermediates, we calculated the reaction enthalpies of the Volmer step on the surface (H_2_O_ads_ → H_ads_ + OH, see Table 1). In many studies, the Volmer step is considered the more unfavorable step of the Volmer–Heyrovsky or Volmer–Tafel mechanisms because hydrogen must be abstracted from water.^4,17,18^ On Pt–Ni, the (100) facet most energetically favors water-splitting at both the lattices of 3.77 and 3.92 Å, followed by the (111) facet at the lattice of 3.77 Å. Whereas the Pt–Ni(110) facet does energetically favor water-splitting at the compressed lattice of 3.62 Å, this facet and lattice also display stronger binding of H and OH and may be less likely to desorb these intermediates and free up active sites. In contrast, the (100) and (111) facets of Pt–Ni clearly display catalytically advantageous properties: weakened H and OH binding for favorable desorption of these species and smaller energetic barriers to water-splitting at the Volmer step.

**Table 1:** Reaction Enthalpies of the HER at the Volmer Step

system	lattice constant (Å)	H_2_O_ads_ → H_ads_ + OH Δ*H*_rxn_ (eV)
Pt(100)	3.92	2.96
	3.62	4.24
Pt-skin on Ni_3_Pt(100)	3.77	2.96
	3.92	2.95
Pt(110)	3.92	3.13
	3.62	2.99
Pt-skin on Ni_3_Pt(110)	3.77	3.13
	3.92	3.18
Pt(111)	3.92	3.07
	3.62	3.99
Pt-skin on Ni_3_Pt(111)	3.77	3.01
	3.92	3.05

**Figure 5:**
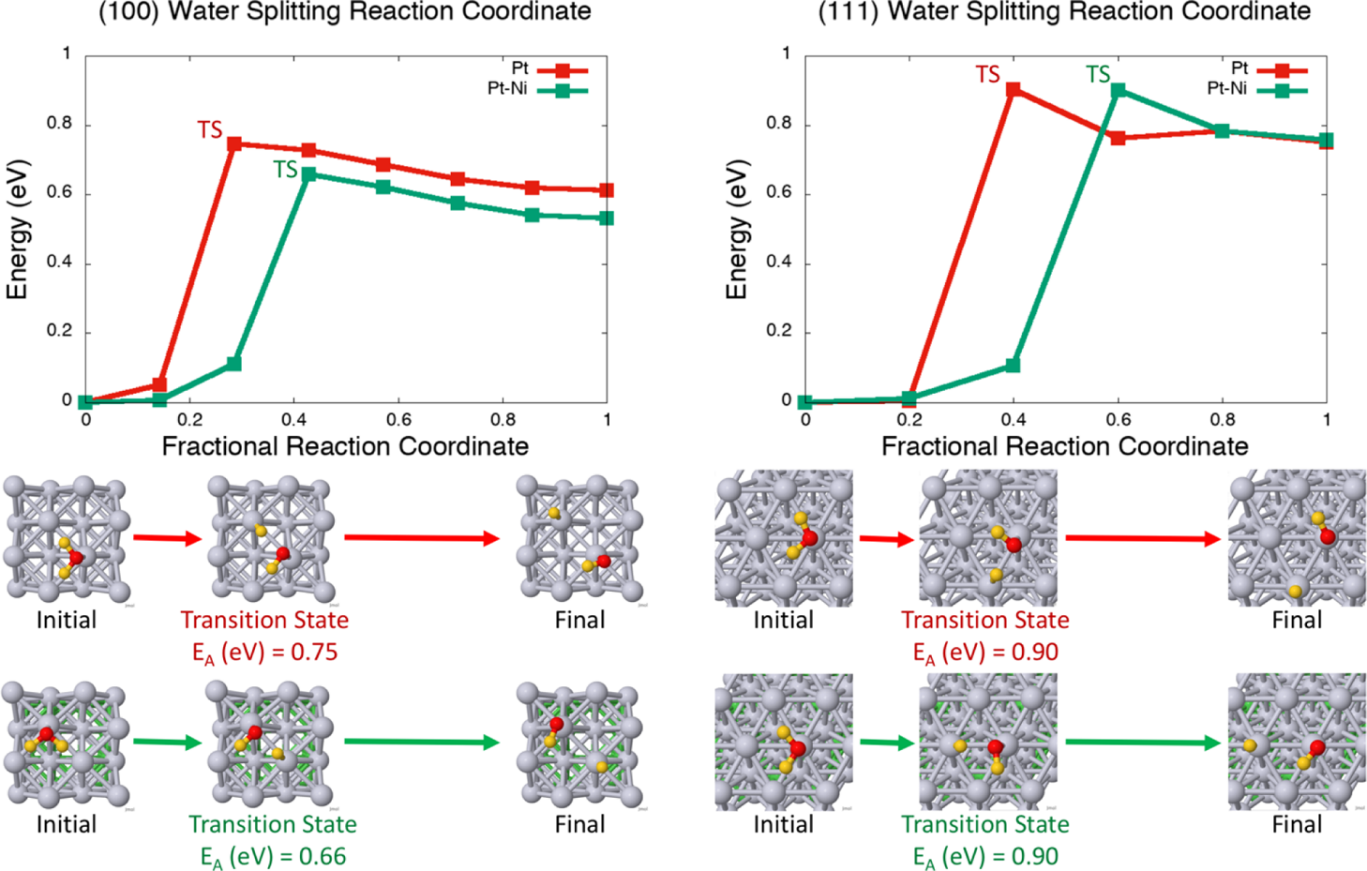
cNEB calculations of the water-splitting reaction of the pure Pt surface (red) *vs* the Pt–Ni surface (green) at a lattice constant of 3.77 Å for the (100) (left) and (111) facets (right). The mechanistic pathway is visualized below the plot of the reaction coordinate, summarizing the initial/final states, and activation energy (*E*_A_) at the transition state (the highest point in the barrier calculation) is displayed. Pt atoms are in gray, Ni atoms are in green, O atoms are in red, and H atoms are in yellow.

**Figure 6:**
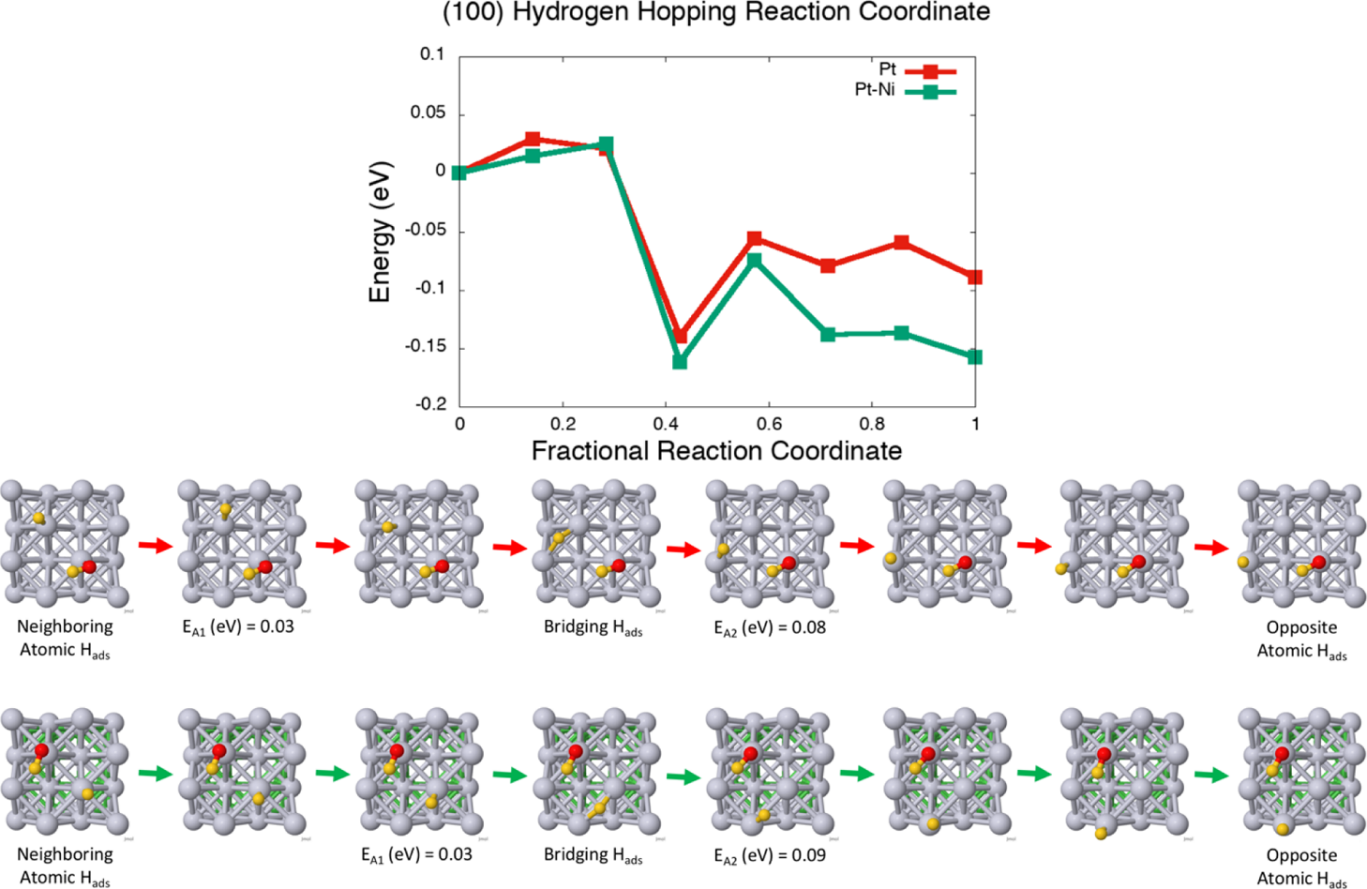
cNEB calculations of the proton-hopping mechanism on the (100) facet of the pure Pt surface (red) *vs* the Pt–Ni surface at a lattice constant of 3.77 Å (green). The mechanistic pathway is visualized below the plot of the reaction coordinate, summarizing different key points: initial/final states, activation energy (*E*_A_) at the transition state (the highest point in the barrier calculation), and various sites that hydrogen can hop to. Pt atoms are in gray, Ni atoms are in green, O atoms are in red, and H atoms are in yellow.

This led us to explore in greater detail the mechanism of water-splitting on the pure Pt surface in comparison to the Pt–Ni surface at a lattice constant of 3.77 Å, focusing particularly on the (100) and (111) facets. The cNEB method was utilized to perform barrier calculations, and the results are displayed in Figure 5.^67^ On the (100) facet, the rate-determining step is of hydrogen splitting from the water to adsorb to the nearest neighboring metal atom. From that metal atom, the hydrogen can hop to farther, but more stable, adsorption sites such as a bridging site or an atomic site opposite of the water (Figure 6). The barrier to this is <0.10 eV and contributes to freeing up the active metal site. On the (100) surface, the barrier to water-splitting is lowered from the pure Pt surface to the Pt–Ni surface by ∼0.10 eV. On the (111) facet, we considered the two unique, nearest neighboring metal sites on the (111) facet; these pathways resulted in an activation energy of 0.90–0.91 eV; the lowest energy pathways are visualized in Figure 5 (alternative pathways are visualized in Supporting Information, Figures S10 and S11). In contrast to the (100) facet, the barrier to water-splitting on the (111) facet is the same on the pure Pt surface and on the Pt–Ni surface, requiring higher activation energy of 0.90 eV. This activation energy of 0.90 eV on the (111) facet is considerably higher than the (100) Pt–Ni surface’s activation energy of 0.66 eV.

The (100) facet of Pt–Ni will favor water-splitting at the Volmer step of HER; following water-splitting, however, the complex relationship between the coadsorbates H_ads_ and OH_ads_ may influence H_2_ formation *via* the Heyrovsky or Tafel mechanism. The mobility of the single H_ads_ becomes important to allowing H_2_ formation to occur. While the (100) facet may bind hydrogen stronger than the (111) facet, the barrier to hydrogen hopping from site to site is minimal at <0.10 eV. Hydrogen will also preferentially hop farther away from OH_ads_, possibly discouraging the reverse reaction of water formation. In contrast to the (100) facet, the (111) facet displays weaker H and OH binding. Moreover, OH binding on the (111) facet is usually weaker than H binding on both the pure Pt surface and also on the Pt–Ni surface at the lattice constants of 3.77 and 3.92 Å. Whereas the (111) facets may feature higher barrier to water-splitting, the (111) facets will also more easily desorb OH and more favorably adsorb H to active sites. We note that our calculations occur in vacuum and neglect the complexity of solvation and the potential gradient present at the surface. However, our detailed discussion of the binding of key adsorbates and the interplay of possible mechanisms to the HER does indicate different activity trends for the (100) facet *versus* the (111) facet. Our calculations may explain the differences in activity of the Pt–Ni catalysts, with a majority {100} exposed, which exploit the lowered activation energy to water-splitting, over pure Pt with a majority of {111}. We note that the facet dependence of activity has been observed experimentally in acidic media for the oxygen reduction reaction and for HOR–HER, where the activity trend was often (110) > (100) > (111) for pure Pt surfaces.^64,68^

### Material Optimization and MEA Testing

Although hydrogen-annealed Pt–Ni nanowires produced high HER activity in RDE half-cells, their MEA performance in single-cell electrolyzers was limited. The disparity between RDE and MEA testing was likely due to the role of RDE conditioning, where potential cycling in acid removed Ni at or near the nanowire surface, exposed Pt sites, and increased activity (Figure S12). Conversely, the potentials and pH of MEA conditioning in AEM electrolysis likely prevented Ni dissolution.^4^ To improve MEA implementation of the nanowires, the hydrogen annealed high performer (275 °C) was exposed to dilute acid (0.05 M nitric acid) to remove near-surface Ni, expose Pt, and improve MEA performance. Following acid exposure, the Pt–Ni nanowires were characterized *ex situ* and in RDE half-cells.

In *ex situ* characterization, several differences were found. The acid leached nanowires were 10.1 wt % Pt, indicating that a small amount of Ni was removed during the exposure to nitric acid. XRD analysis revealed that some Pt lattice expansion occurred and that the pattern was more similar to the annealed nanowires following electrochemical conditioning (Figure 3b,c). The XRD pattern also did not significantly change following electrochemical conditioning, indicating that potential cycling was less critical for HER–HOR performance and that the acid leached material may be better suited for MEA implementation (confirmed in RDE, Figure S13).

**Figure 7:**
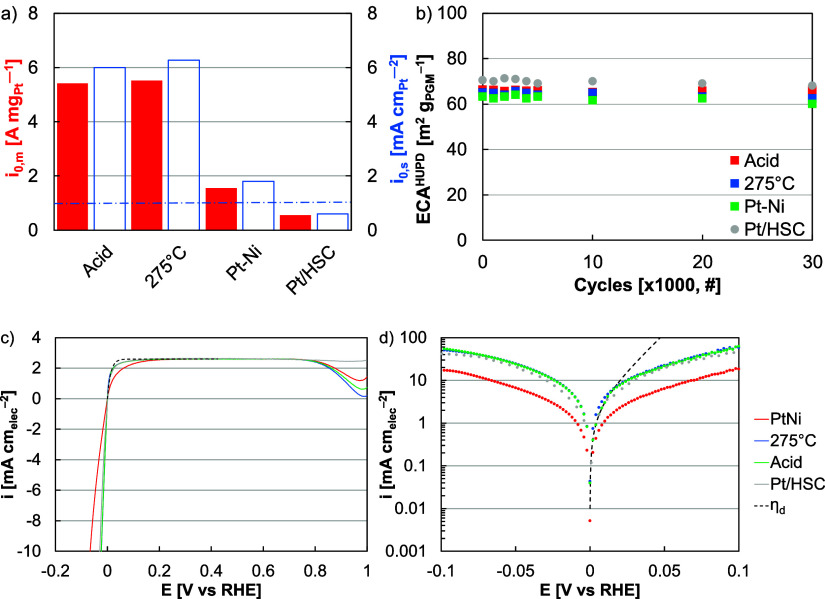
(a) HER–HOR mass (red) and site-specific (blue) exchange current densities of as-synthesized (Pt–Ni), hydrogen annealed (275 °C), and acid leached (Acid) Pt–Ni nanowires and Pt/HSC in RDE half-cells. The site-specific (dashed blue) activity of polycrystalline Pt is provided as a horizontal line. (b) Pt ECAs of as-synthesized (Pt–Ni), hydrogen annealed (275 °C), and acid leached (Acid) Pt–Ni nanowires and Pt/HSC as a function of cycles in the potential range −0.2 to 0.2 V *versus* RHE. (c) Linear sweep voltammograms and (d) Butler–Volmer plots of as-synthesized (Pt–Ni), hydrogen annealed (275 °C), and acid leached (Acid) Pt–Ni nanowires and Pt/HSC with the Nernstian diffusion limited overpotential (η_d_, dashed line).

The acid leached catalyst produced an HER–HOR activity similar to the hydrogen-annealed sample in RDE testing. A marginally lower activity (2% less) was due to lower site-specific activity and may have been affected by slight differences in: lattice, with the average Pt lattice spacing of the hydrogen annealed and acid leached catalysts at 3.79 and 3.80 Å, respectively (Figure 3b,c); facet, with acid leaching resulting in higher prevalence of exposed Pt{100} (Figures 3d,e, S14 and S15); and near-surface Ni, with acid leaching removing Ni that may have aided activity by providing oxophilic centers near Pt sites.^16,25,63^ The acid leached catalyst produced a HER–HOR mass exchange current density of 5.4 A mg_Pt_^–1^, approximately 2% less than the hydrogen annealed nanowires, more than 3 times the as-synthesized nanowires (3.5 times), and more than 9 times greater than Pt/HSC (9.8 times, Figures 7a,c,d and S16).

Additionally, Pt–Ni nanowire durability was evaluated in RDE half-cells by potential cycling (30,000 cycles) in the range of −0.2 to 0.2 V in a hydrogen saturated electrolyte (Figures 7b, S17 and S18). These potentials were chosen to include the widest anticipated operating range, whether being used in electrolyzers, fuel cells, or reversible fuel cells. Appreciable loss in activity or ECA was not found for any of the catalysts evaluated, including the acid leached, hydrogen annealed, and as-synthesized nanowires and Pt/HSC (Figures 7b, S17 and S18). Loss was not expected from Pt-based catalysts because Pt redox and dissolution occur at much higher potentials.^4^ The result, however, confirmed that the presence of Ni did not adversely impact durability because of oxide growth or aggregation. The nanowires may have mitigated durability concerns because the approximate Pt coatings minimized the Ni–electrolyte contact and the nanowire size/morphology minimized aggregation-based loss.

**Figure 8:**
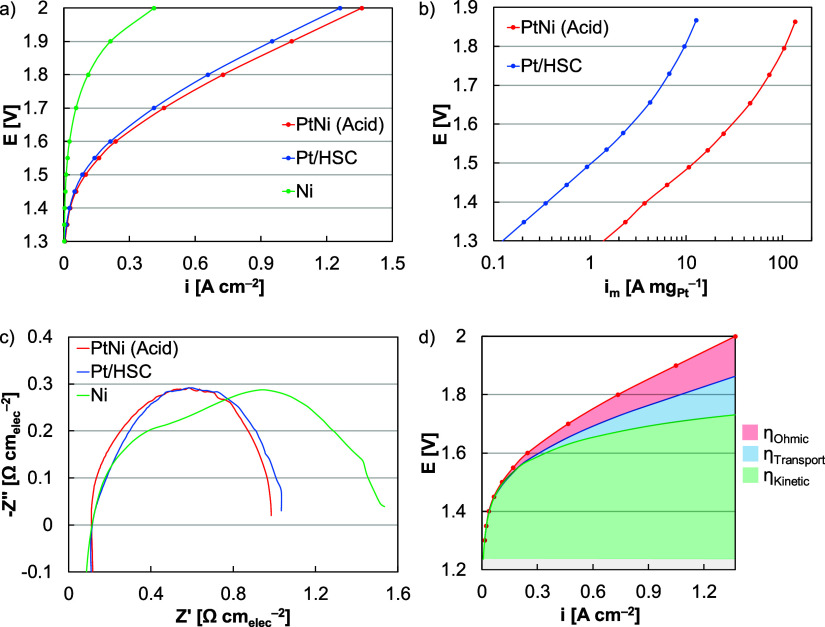
(a) MEA polarization curves, (b) Tafel plots, and (c) impedance data of acid leached Pt–Ni nanowires, Pt/HSC, and Ni nanoparticles. MEAs were tested in electrolysis mode with Co anodes (0.4 mg_Co_ cm^–2^), NREL Gen 2 perfluorinated AEMs and ionomers (ionomer to catalyst ratio of 0.22), Toray transport layers, and Ni flow fields. Cathode catalyst layers were sprayed to loadings of 0.1 (Pt–Ni, Pt/HSC) and 0.2 (Ni) mg_M_ cm^–2^. Tafel plots in (b) were corrected for high frequency resistance and impedance experiments in (c) were completed at 0.01 A cm^–2^. (d) Breakdown of Ohmic, transport, and kinetic losses for the acid leached Pt–Ni nanowire MEA.

In single-cell MEAs, testing was completed using a perfluorinated AEM and ionomer (NREL Gen 2), a cobalt anode (ionomer/catalyst ratio of 0.22:1, loading of 0.4 mg_Co_ cm^–2^), and a variable cathode (Pt/HSC, Pt–Ni, Ni, Figure 8a). Significant differences were noted between AEM-MEA testing and typical PEM electrolyzer operation that impacted the losses and the relative role of kinetics in MEA performance (Figure 8d). First, the AEMs were approximately 50 μm thick and resulted in lower membrane resistance and lower Ohmic losses (Figure 8c). Second, catalyst layers were sprayed onto PTLs as opposed to directly spraying catalyst-coated membranes; this was necessary to prevent immediate performance deterioration and may have resulted in higher transport or Ohmic losses and lower catalyst site access. Within the kinetic region, the Pt–Ni nanowire MEA outperformed Ni nanoparticles by 20 times and Pt/HSC by 5%, similar to performance differences in RDE half-cells (Figure 8b). Compared to the Pt baseline, the nanowires kinetically produced an order of magnitude higher performance on a Pt basis; in terms of cell performance, the optimized nanowires produced a similar current density at a PGM loading one tenth of Pt/HSC. Differences in kinetic performance between the nanowires and Pt/HSC generally translated to higher current density; this gap may vary under different test conditions, including other membrane/ionomer combinations and cell configurations (catalyst-coated membranes). In extended operation, a potential hold at 2 V revealed performance losses in MEAs with Pt–Ni and Pt/HSC cathodes (Figure S19). The losses, however, were similar and likely due to oxide growth in the anode catalyst layer (Co); several other factors, including flow field (Ni) oxide growth, PTL corrosion, electrolyte carbonation, and changes in the catalyst layer (ionomer integration) and interface (membrane), may have contributed. Although Pt-cathode loss was not expected due to potential requirements, these results suggested that the Ni template was not detrimental (oxide growth) to AEM electrolysis operation.

## Conclusions

Pt–Ni nanowires were developed as hydrogen evolving electrocatalysts for AEM-based electrolyzers. Synthesis by galvanic displacement produced Pt-coated nanowires with high surface areas and activities that may have benefitted from a high proportion of Pt{100} surfaces and avoiding fringe facets. Hydrogen annealing significantly improved specific activity, and the optimum catalyst was more active than the as-synthesized nanowires and the nanoparticle baseline by 3 and 10 times, respectively. Activity improvements were likely due to weakened intermediate binding of H and OH from Pt lattice compression and lowered activation energy to water-splitting, specifically on the (100) facet, relative to the uncompressed (Pt–Ni system) and pure Pt surface. Barrier calculations at the potentially rate-limiting Volmer step were pursued on the (100) and (111) facets, comparing the water-splitting reaction on the compressed lattice of 3.77 Å on Pt–Ni to the pure-Pt surface. The (100) facet featured lower activation energies of 0.66 eV on Pt–Ni and 0.75 eV on Pt, whereas the (111) facet remained high at 0.90 eV on both Pt–Ni and pure Pt. However, the (111) facet often features stronger H adsorption than OH adsorption, which may be advantageous in alkaline media, reserving active sites for H over OH. This has particular implications for the Heyrovsky and Tafel steps to H_2_ formation. Acid leaching was used to remove near-surface Ni and incorporates the nanowires into electrolyzer MEAs, where the nanowire performance was kinetically 1–2 orders of magnitude greater than Ni and slightly better than Pt nanoparticles while at one tenth the Pt loading.

While electricity cost drives the price of hydrogen produced by electrolysis today, capital and catalyst costs will become increasingly critical as electrolyzers are directly paired with low-cost, renewable power sources. AEM systems offer several advantages to PEM-based electrolyzers, including the ability to use non-PGMs as catalysts (particularly at the anode), as component coatings (transport layers and separators), and the improved durability of materials at high pH. The Pt–Ni nanowires developed in this study produced high levels of activity in half- and single-cell tests. The nanowire HER activity was significantly higher than Ni while exceeding Pt performance at one tenth the loading. Theoretical calculations identified the facet and lattice dependence of the water-splitting reaction, finding that the activation energy is significantly lowered on the compressed (100) Pt–Ni surface by circa 0.2 eV as compared to the (111) surfaces of Pt and Pt–Ni. Therefore, nanowires with a high proportion of Pt{100} may be particularly active as compared to catalysts featuring only Pt{111}. The testing of these materials demonstrates that PGM loading reductions are possible in electrolysis systems without losing performance. These catalysts provide a low-PGM catalysis option in HER and can potentially enable the use of renewable hydrogen producing systems.

## Supporting Information Available

High-angle annular dark-field imaging, high-angle annular bright-field imaging, and EDS of Pt−Ni nanowires (as-synthesized and hydrogen annealed to 275 °C); germanium and tellurium adsorption/desorption; adsorption energies of H_ads_ and OH_ads_; adsorption sites of global minimum structures of H_ads_, OH_ads_, and H_2_O_ads_; global minimum structures of adsorbed H, OH, and H_2_O on Pt surfaces, Pt-Ni (100) surfaces, Pt-Ni (110) surfaces, and Pt-Ni (111) surfaces; cNEB calculations; HER−HOR activities and cyclic voltammograms during conditioning (as-synthesized and acid leached); linear sweep voltammograms, Butler-Volmer plots, and HER−HOR activities of examined catalysts (prior to and following durability testing); equivalent circuit model fits; and polarization curves of MEAs during extended operation (PDF)

## Abbreviations

Pt–Ni: platinum–nickel
AEM: anion exchange membrane
PGM: platinum group metal
HER: hydrogen evolution reaction
Pt: platinum
HOR: hydrogen oxidation reaction
Ni: nickel
RDE: rotating disk electrode
MEA: membrane electrode assembly
ICP–MS: inductively coupled plasma–mass spectrometry
XRD: X-ray diffraction
TEM: transmission electron microscopy
STEM: scanning transmission electron microscopy
EDS: energy dispersive spectroscopy
Pt/HSC: Pt supported on high surface area carbon
RHE: reversible hydrogen electrode
ECA: electrochemical surface area
PTL: porous transport layer
Co: cobalt
cNEB: climbing image nudge-elastic band
*E*_A_: activation energy
