# Supplementary material for: Platinum–Nickel Nanowires with Improved Hydrogen Evolution Performance in Anion Exchange Membrane-Based Electrolysis
Source: ACS Catal. 2020 Jul 7;10(17):9953–66. doi: 10.1021/acscatal.0c01568 (PMC10906943; doi:10.1021/acscatal.0c01568)
Supplement: Supplementary file 1 — cs0c01568_si_001.pdf [file cs0c01568_si_001.pdf]

Supporting Information

Platinum-Nickel Nanowires with Improved Hydrogen Evolution Performance in Anion Exchange Membrane-Based Electrolysis

Shaun M. Alia [1,†,], Mai-Anh Ha [2,†], Chilan Ngo [3], Grace C. Anderson [1], Shraboni Ghoshal [1], and Svitlana Pylypenko [3]*

[1] Chemistry and Nanoscience Center, National Renewable Energy Laboratory, 15013 Denver West Parkway, Golden, CO 80401, United States

[2] Computational Science Center, National Renewable Energy Laboratory, 15013 Denver West Parkway, Golden, CO 80401

[3] Department of Chemistry, Colorado School of Mines, 1012 14th Street, Golden, Colorado 80401, United States

[†] These authors contributed equally to this work

* Corresponding author, Shaun M. Alia, shaun.alia@nrel.gov

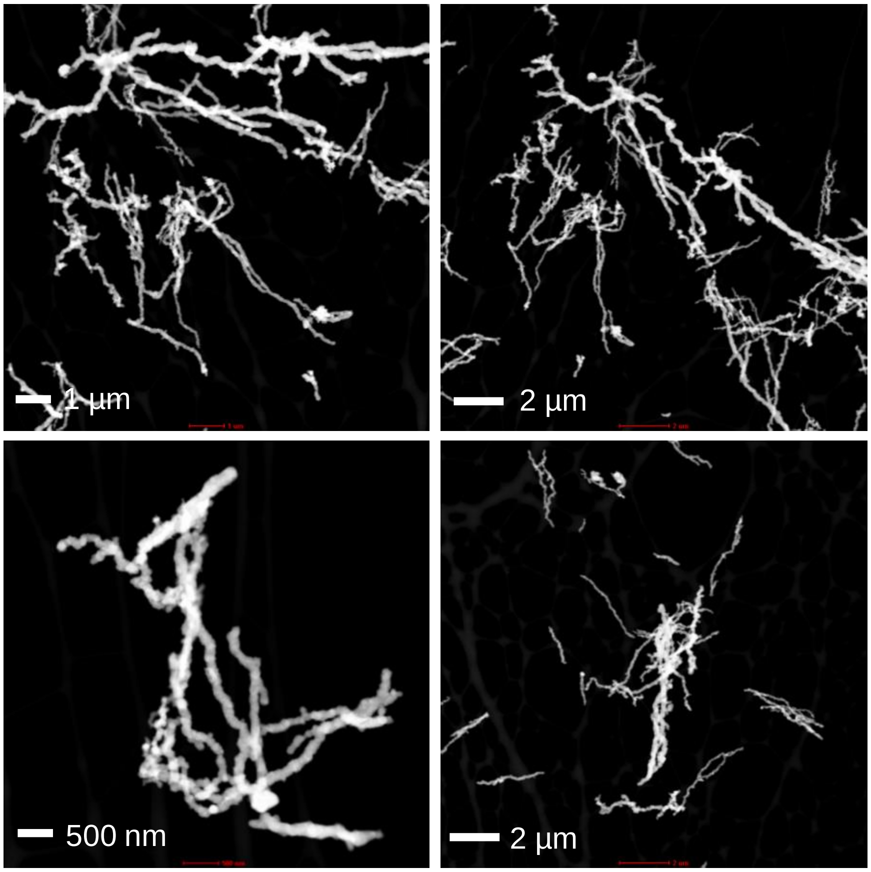

Figure S1. High-angle annular dark-field imaging of Pt-Ni nanowires, 7.3 wt. % Pt and annealed to 275°C. Images were used to evaluate nanowire length.

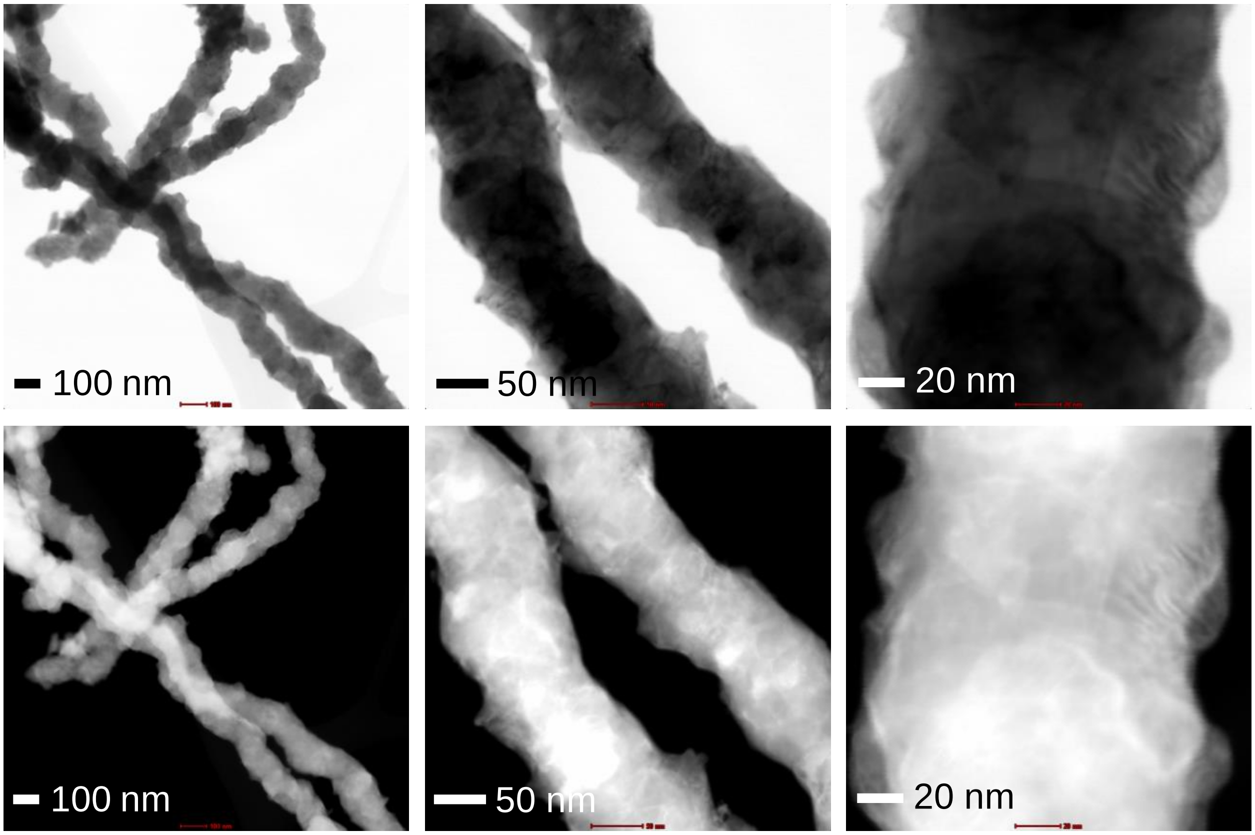

Figure S2. High-angle annular bright-field (top row) and dark-field (bottom row) imaging of Pt-Ni nanowires, 7.3 wt. % Pt and annealed to 275°C. Images were used to evaluate nanowire diameter.

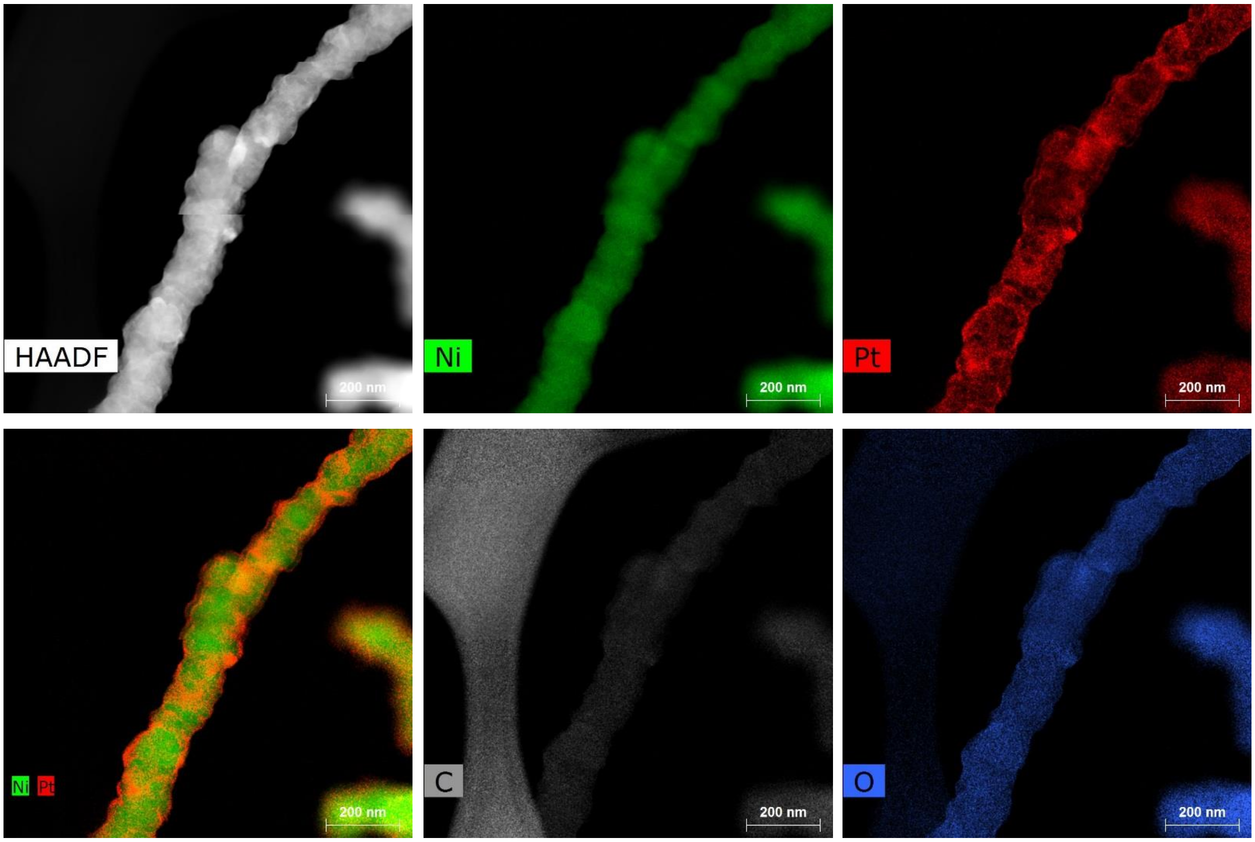

Figure S3. High-angle annular dark-field imaging and energy dispersive x-ray spectroscopy of Pt-Ni nanowires, 7.3 wt. % Pt and annealed to 275°C. Images were used to evaluate the Pt coating.

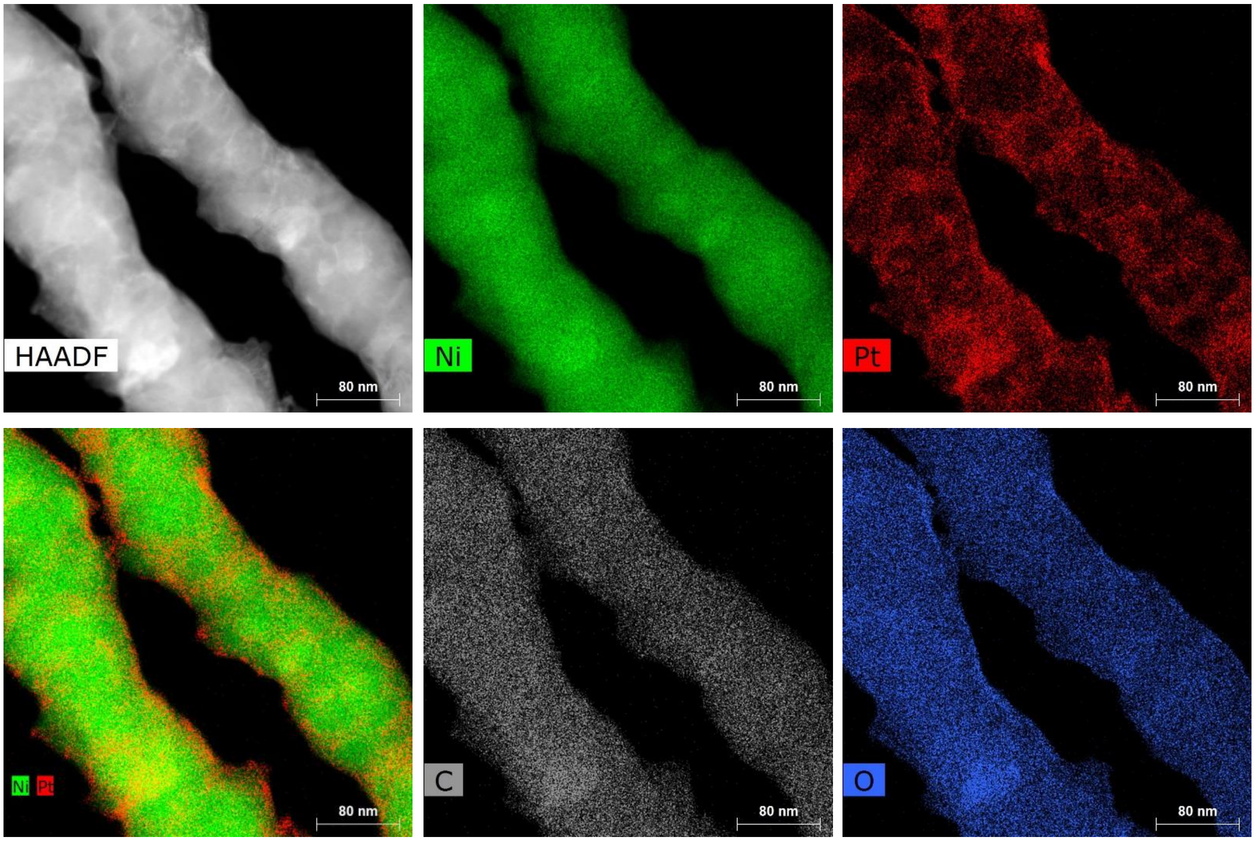

Figure S4. High-angle annular dark-field imaging and energy dispersive x-ray spectroscopy of Pt-Ni nanowires, 7.3 wt. % Pt and annealed to 275°C. Images were used to evaluate the Pt coating.

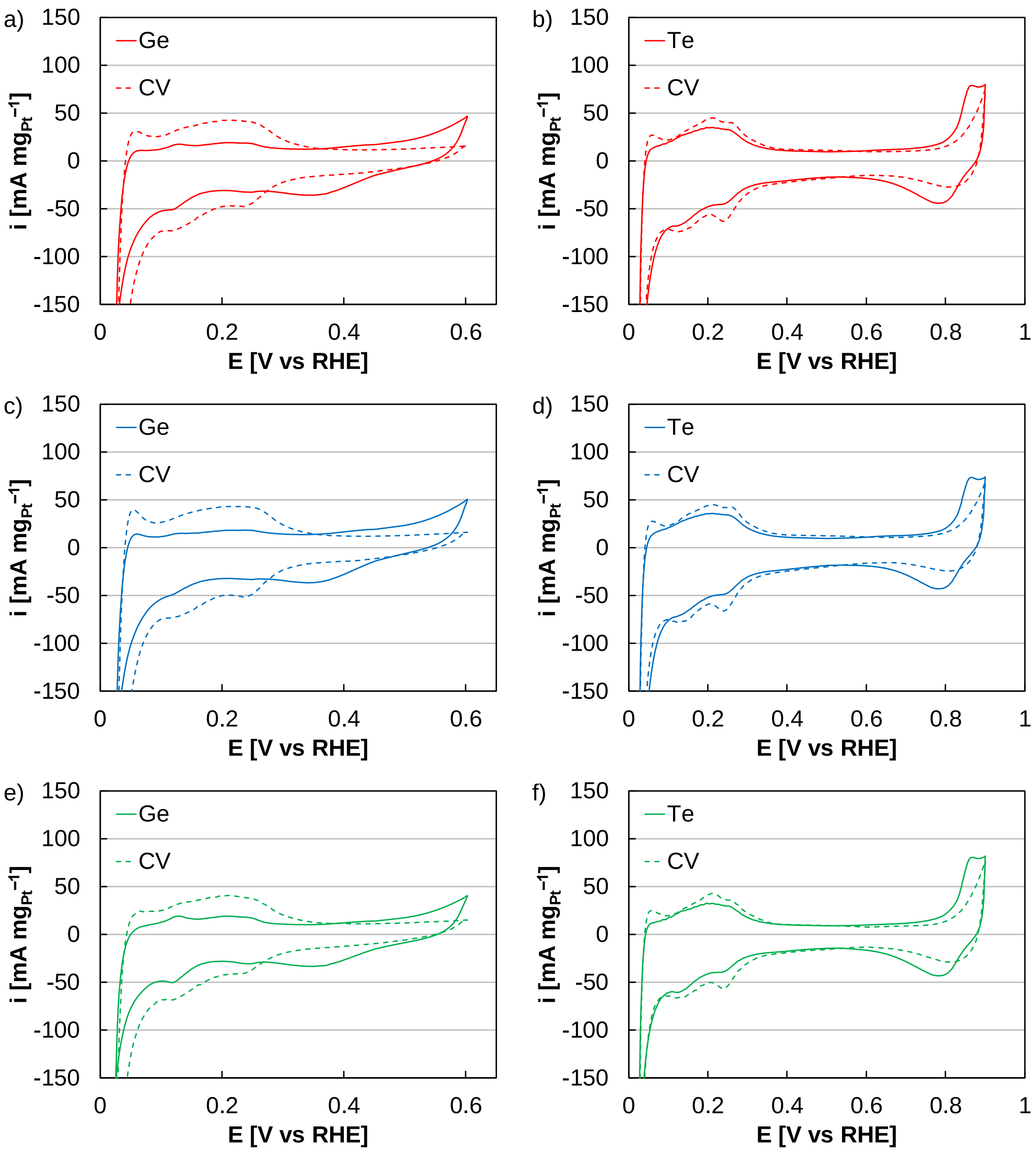

a)
b)
c)
d)
e)
f)
Ge
Te
CV
i [mA mgPt-1]
E [V vs RHE]
150
100
50
0
-50
-100
-150
0
0.2
0.4
0.6
0.8
1

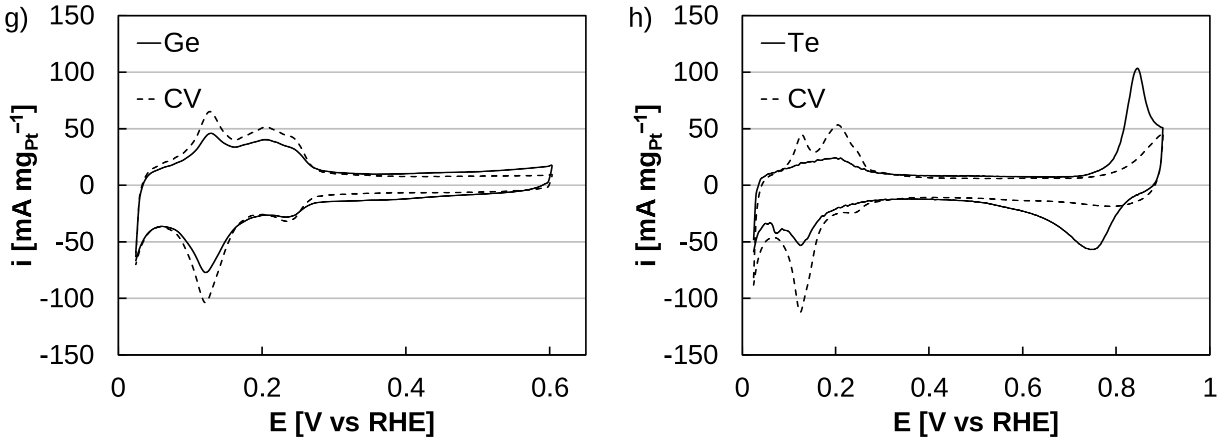

Figure S5. Cyclic voltammograms of (a–b) acid leached Pt-Ni nanowires, (c-d) hydrogen annealed Pt-Ni nanowires (to 275°C), (e–f) as-synthesized Pt-Ni nanowires, and (g–h) Pt/HSC. Voltammograms included electrodes with adsorbed germanium (Ge, left) and tellurium (Te, right), and without adsorbed species (background voltammograms).

Table S1. Adsorption Energies of H_{ads} and OH_{ads}; reaction enthalpies for the Heyrovsky and Tafel mechanisms.

System	Lattice Constant (Å)	H_{ads} (eV)	OH_{ads} (eV)	Heyrovsky: $H_{ads} + H_2O + e^- \rightarrow H_2 + OH^-$ ΔH_{rxn} (eV)	Tafel: $2H_{ads} \rightarrow H_2$ ΔH_{rxn} (eV)
**Pt (100)**	3.92	-2.92	-3.07	3.97	0.65
Pt-skin on Ni_3Pt (100)	3.62	-5.98	-6.86	7.03	3.72
	3.77	-2.89	-2.95	3.94	0.63
	3.92	-2.95	-3.12	4.00	0.68
**Pt (110)**	3.92	-2.87	-2.98	3.92	0.60
Pt-skin on Ni_3Pt (110)	3.62	-3.02	-3.43	4.07	0.75
	3.77	-2.88	-3.11	3.93	0.62
	3.92	-2.84	-2.97	3.89	0.57
**Pt (111)**	3.92	-2.45	-2.12	3.50	0.18
Pt-skin on Ni_3Pt (111)	3.62	-2.05	-2.83	3.10	-0.22
	3.77	-2.74	-2.38	3.79	0.47
	3.92	-2.85	-2.62	3.89	0.58

Table S2. Adsorption Sites of Global Minimum Structures of H_{ads}, $OH_{ads,}$ H_2O_{ads}

System	Lattice Constant (Å)	H_{ads} (eV)	OH_{ads} (eV)	**H_2O_{ads}** (eV)
**Pt (100)**	3.92	Bridging	bridging	atomic
Pt-skin on Ni_3Pt (100)	3.62	Hollow	bridging	atomic
	3.77	bridging	bridging	atomic
	3.92	bridging	bridging	atomic
**Pt (110)**	3.92	bridging	bridging	atomic
Pt-skin on Ni_3Pt (110)	3.62	different bridging from **Pt (110)**	different bridging from **Pt (110)**	atomic
	3.77	bridging	different bridging	atomic
	3.92	bridging	bridging	atomic
**Pt (111)**	3.92	hollow	bridging	atomic
Pt-skin on Ni_3Pt (111)	3.62	bridging	atomic	atomic
	3.77	atomic	atomic	atomic
	3.92	hollow	OH splits: O on hollow, H o atomic	atomic

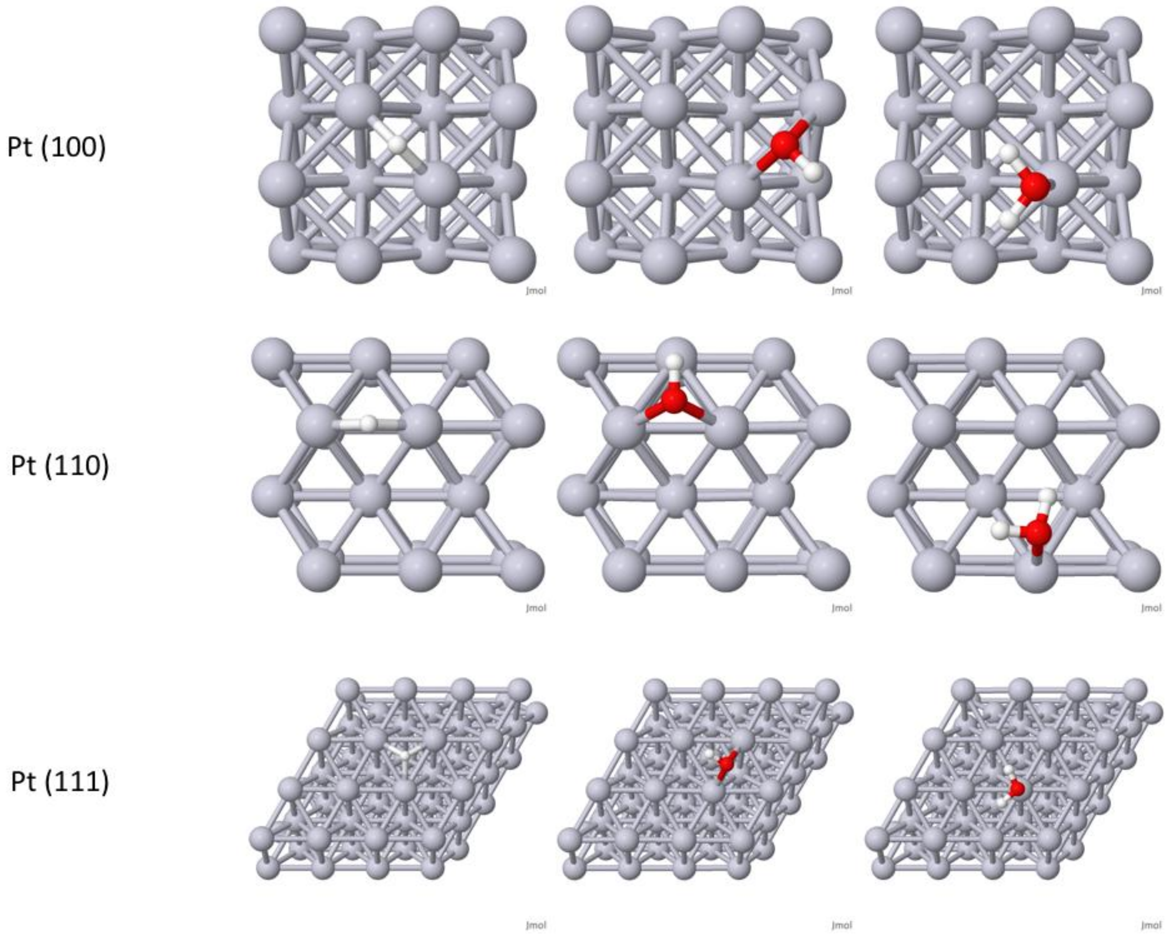

Figure S6. Global minimum structures of adsorbed H, OH, and H_2O on Pt surfaces.

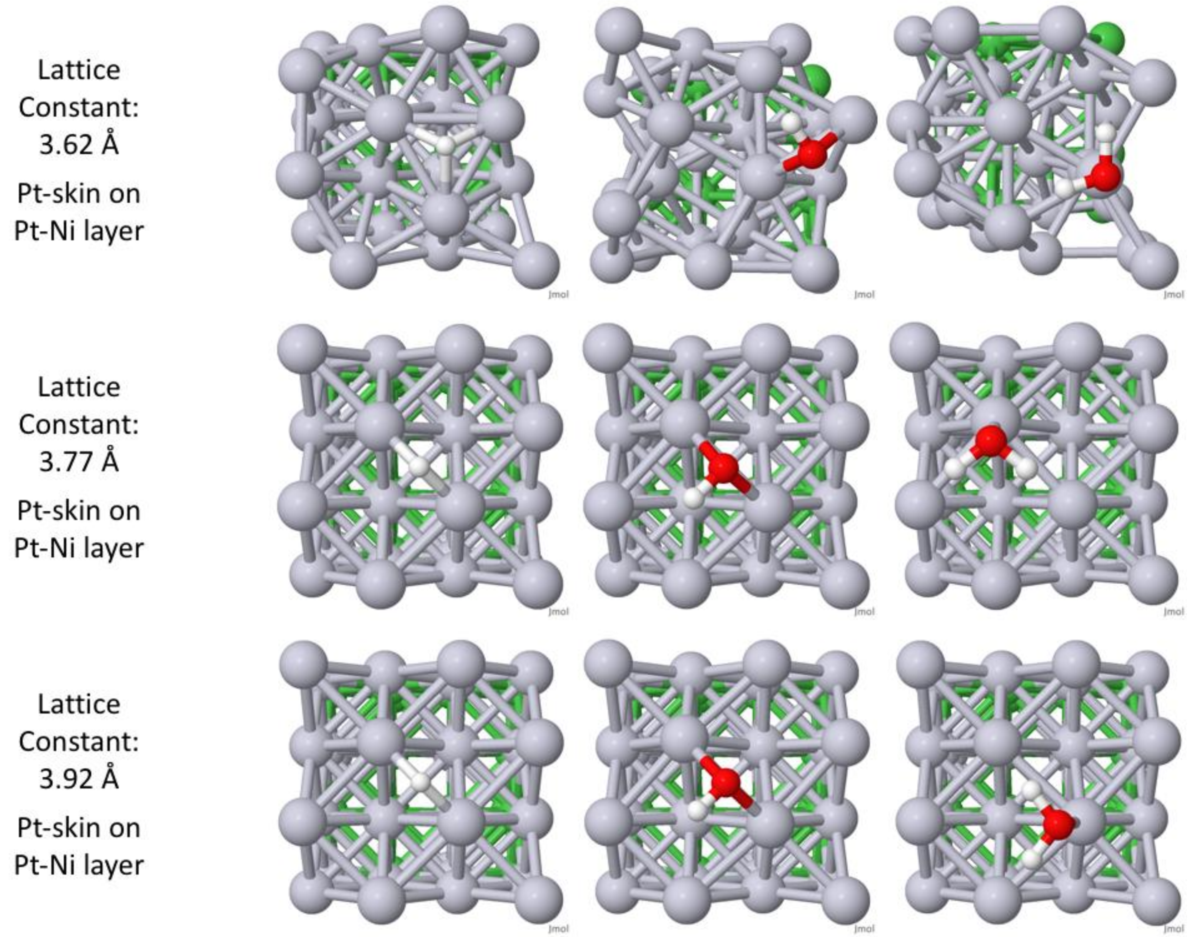

Figure S7. Global minimum structures of adsorbed H, OH, and H_2O on Pt-Ni (100) surfaces.

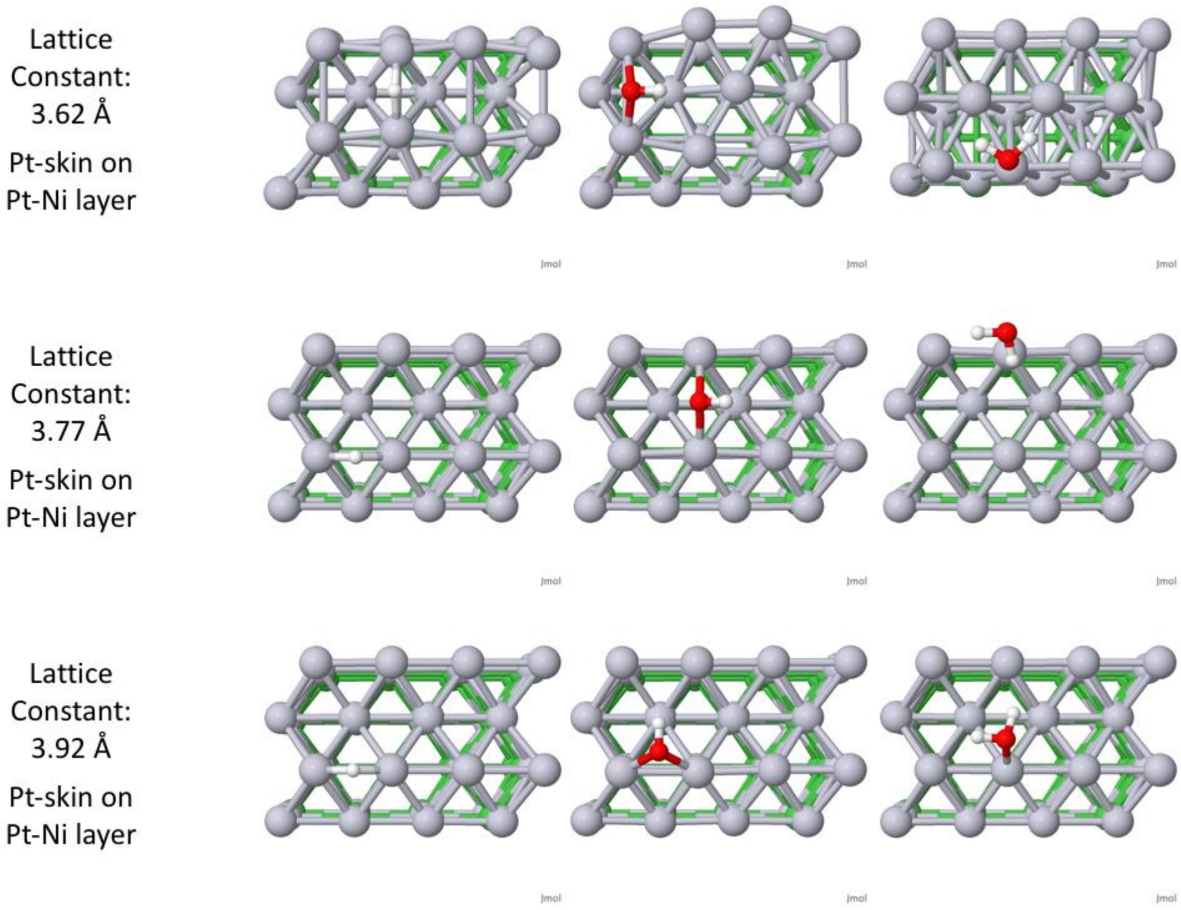

Figure S8. Global minimum structures of adsorbed H, OH, and H_2O on Pt-Ni (110) surfaces.

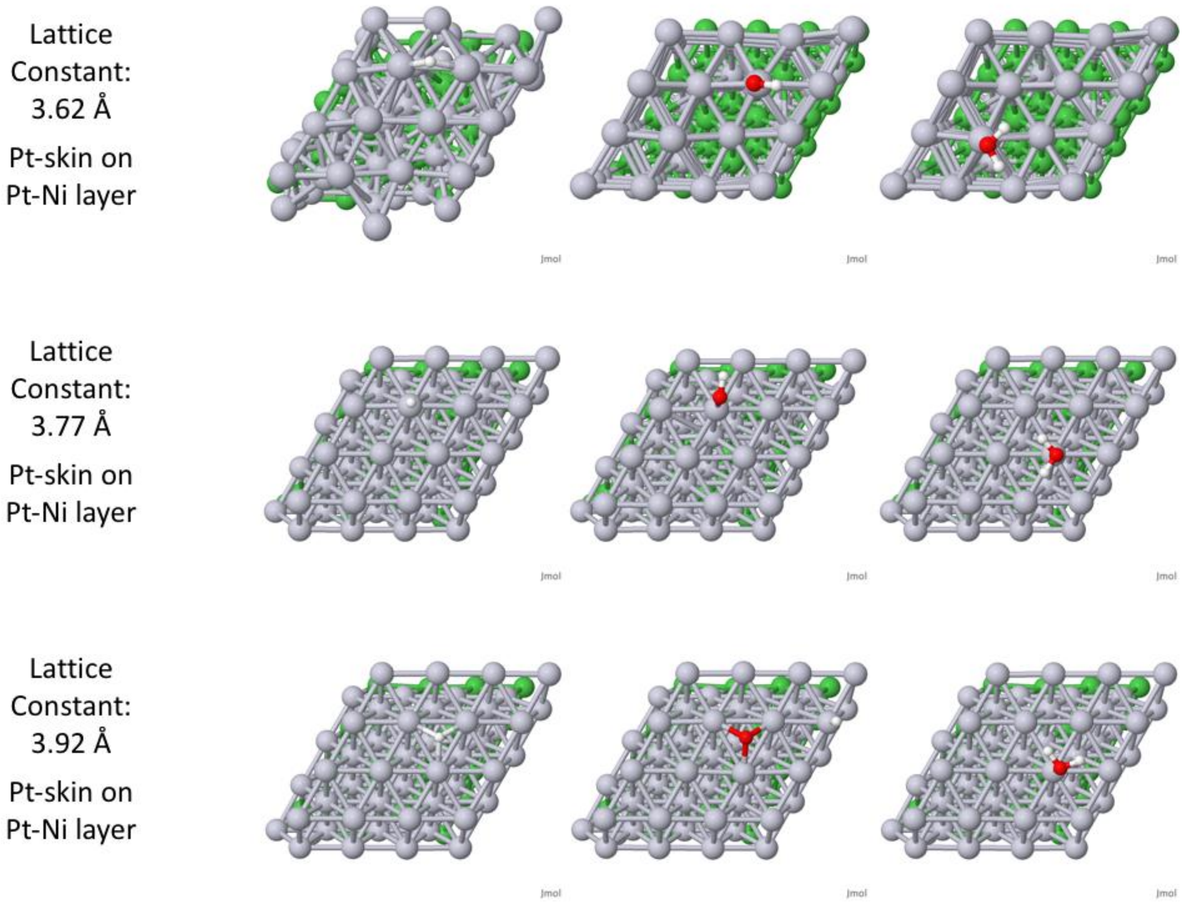

Figure S9. Global minimum structures of adsorbed H, OH, and H_2O on Pt-Ni (111) surfaces.

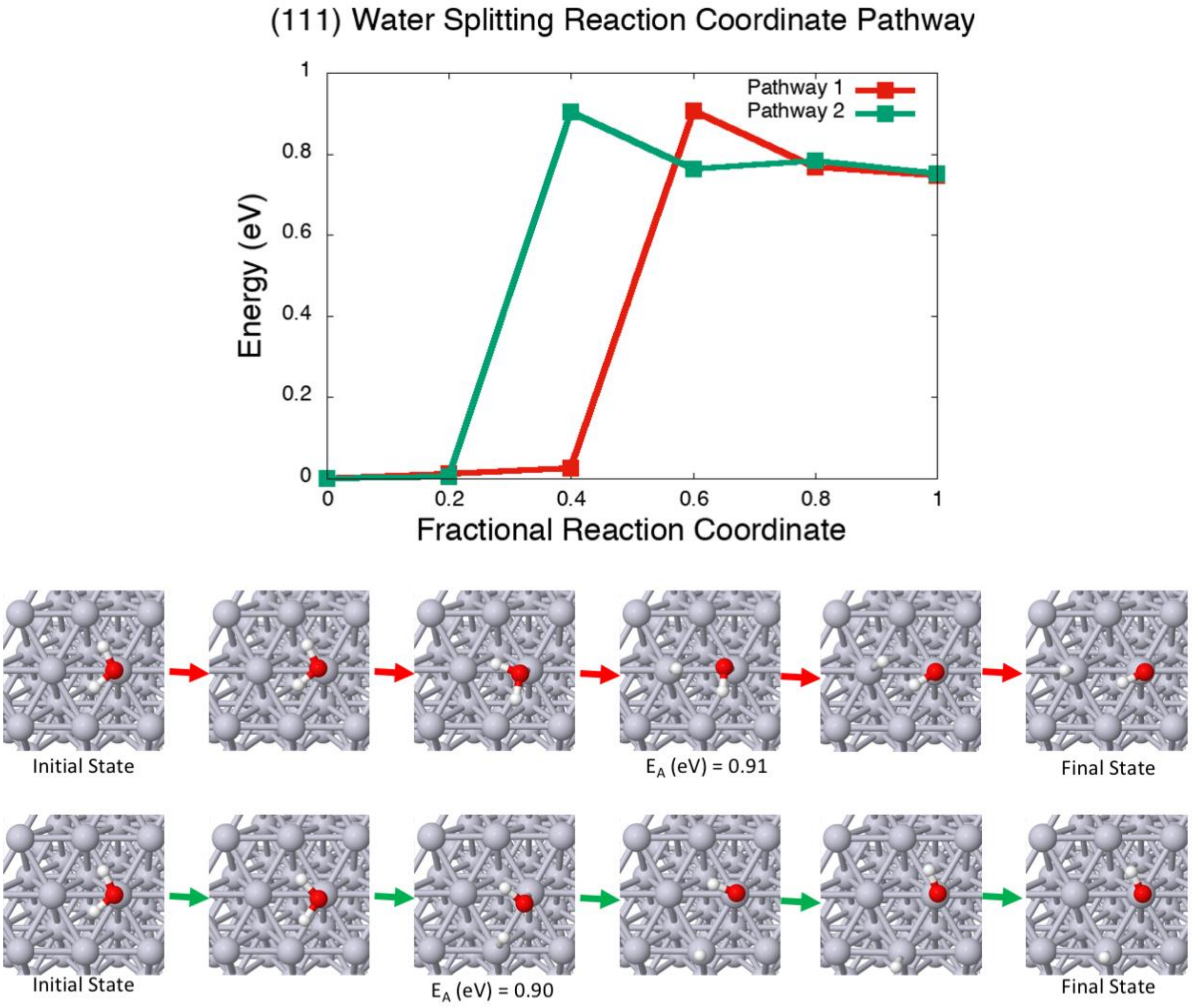

Figure S10. Climbing image nudge elastic band calculations (cNEB) for the different pathways of water-splitting on Pt (111).

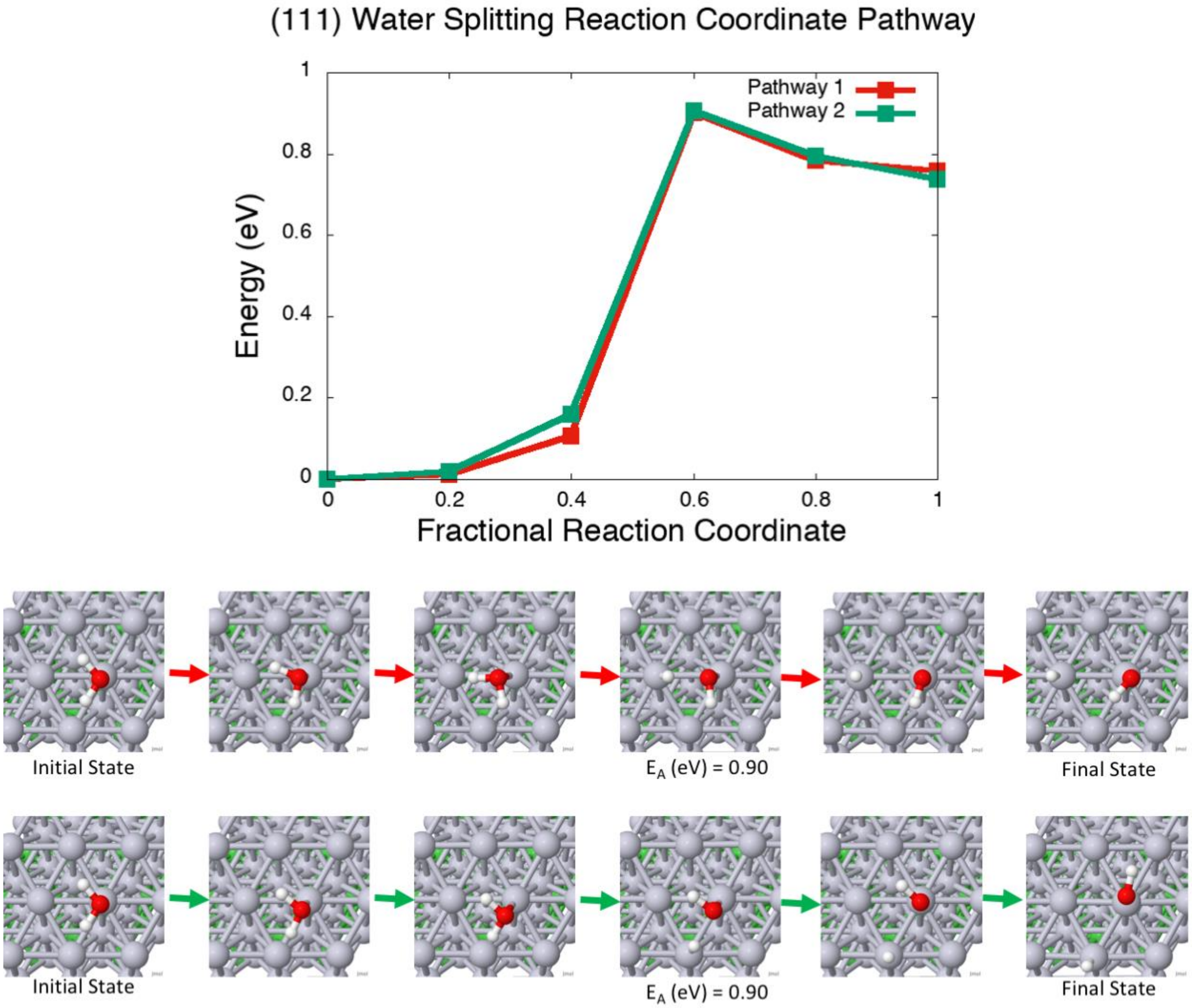

Figure S11. Climbing image nudge elastic band calculations (cNEB) for the different pathways of water-splitting on Pt-Ni (111).

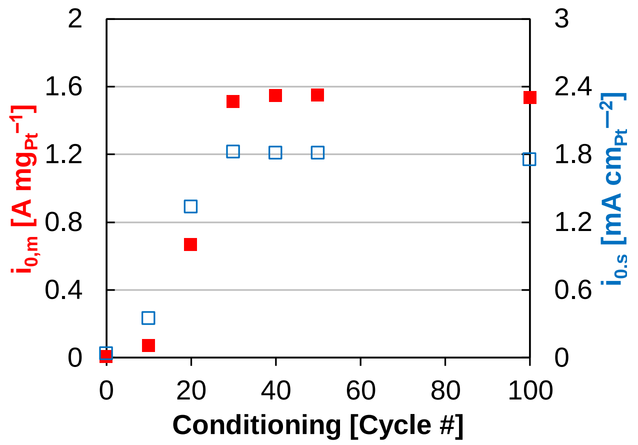

Figure S12. HER-HOR (a) mass (red) and site-specific (blue) exchange current densities of as-synthesized Pt-Ni nanowires in RDE half-cells as a function of conditioning cycles (x-axis). Conditioning was completed in a 0.1 M perchloric acid electrolyte in the potential range 0.025–1.4 V vs RHE. Activities were taken in a 0.1 M sodium hydroxide electrolyte, fit to the Butler-Volmer equation to determine exchange current densities.

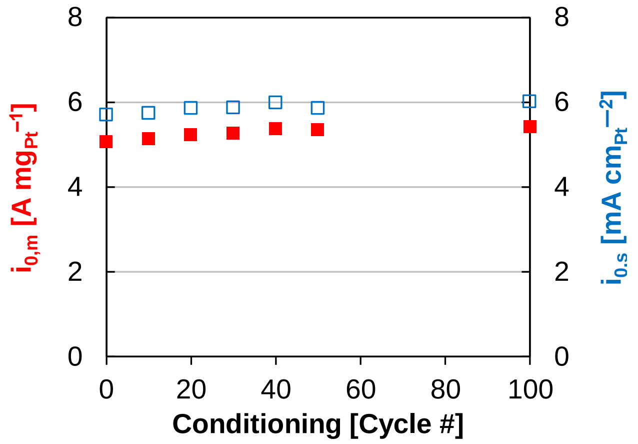

Figure S13. HER-HOR (a) mass (red) and site-specific (blue) exchange current densities of Pt-Ni nanowires, 7.3 wt. % Pt, hydrogen annealed to 275°C, and acid leached in RDE half-cells as a function of conditioning cycles (x-axis). Conditioning was completed in a 0.1 M perchloric acid electrolyte in the potential range 0.025–1.4 V vs RHE. Activities were taken in a 0.1 M sodium hydroxide electrolyte, fit to the Butler-Volmer equation to determine exchange current densities.

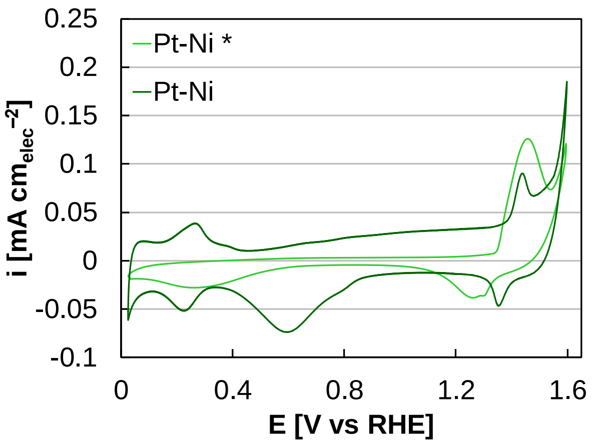

Figure S14. Cyclic voltammograms of as-synthesized Pt-Ni nanowires in a 0.1 M sodium hydroxide electrolyte, prior to (Pt-Ni) and following (Pt-Ni *) conditioning in a 0.1 M perchloric acid electrolyte. Conditioning in the acidic electrolyte consisted of 100 cycles in the potential range 0.025–1.4 V vs RHE at 2500 rpm and 500 mV s^{-1}.

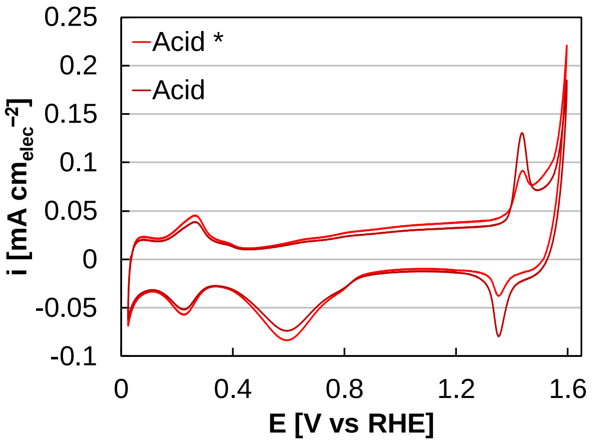

Figure S15. Cyclic voltammograms of acid leached Pt-Ni nanowires in a 0.1 M sodium hydroxide electrolyte, prior to (Acid) and following (Acid *) conditioning in a 0.1 M perchloric acid electrolyte. Conditioning in the acidic electrolyte consisted of 100 cycles in the potential range 0.025–1.4 V vs RHE at 2500 rpm and 500 mV s^{-1}.

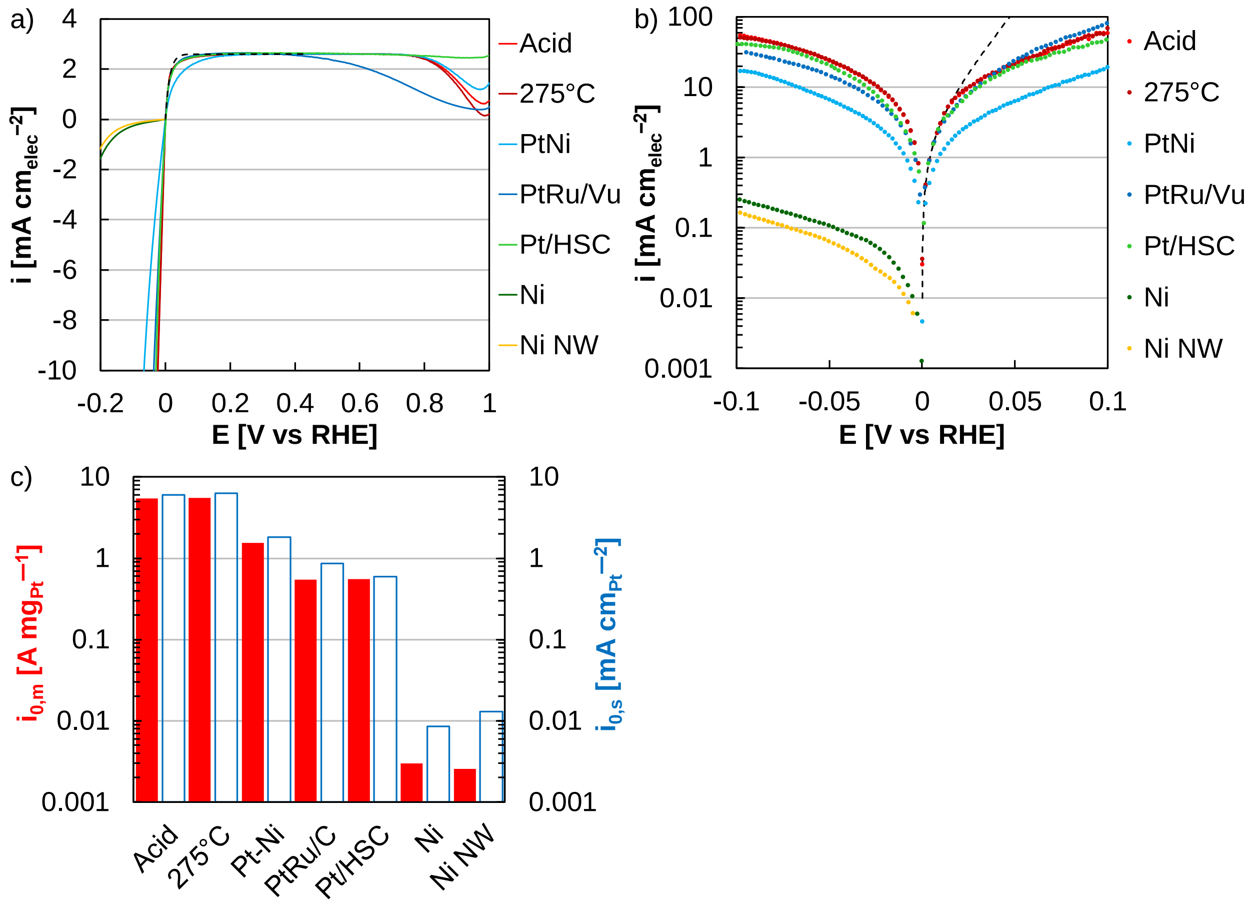

Figure S16. (a) Linear sweep voltammograms and (b) Butler-Volmer plots of acid leached Pt-Ni nanowires (Acid), hydrogen annealed Pt-Ni nanowires (275°C), as-synthesized Pt-Ni nanowires (PtNi), carbon-supported Pt-Ru (PtRu/Vu), carbon-supported Pt (Pt/HSC), Ni nanoparticles (Ni), and Ni nanowires (Ni NW) with the Nernstian diffusion limited overpotential (dashed line). (c) HER-HOR mass (red) and site-specific (blue) exchange current densities of the evaluated catalysts.

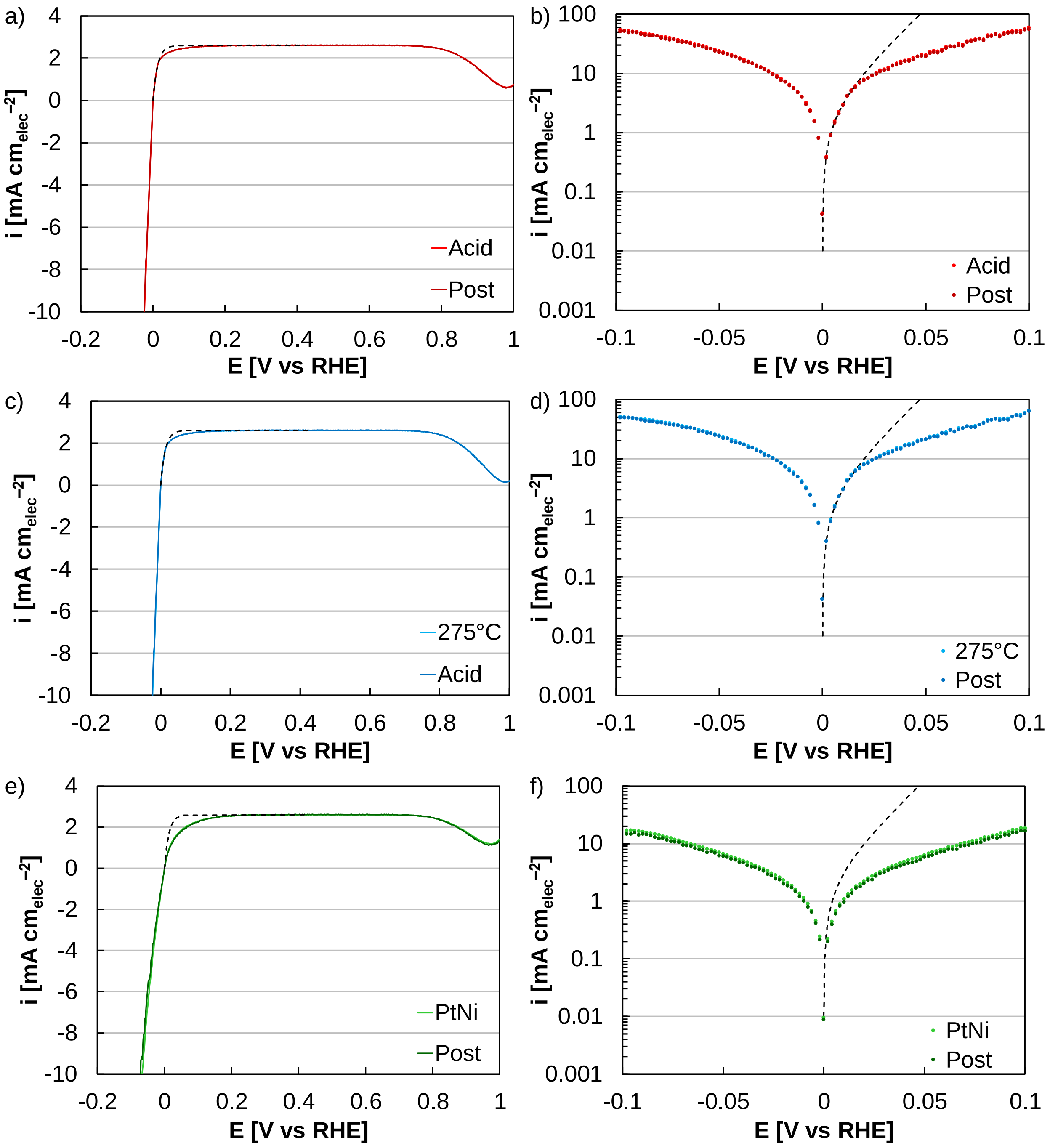
a)
i [mA cm$_{elec}^{-2}$]
E [V vs RHE]
Acid
Post
b)
i [mA cm$_{elec}^{-2}$]
E [V vs RHE]
Acid
Post
c)
i [mA cm$_{elec}^{-2}$]
E [V vs RHE]
275°C
Acid
d)
i [mA cm$_{elec}^{-2}$]
E [V vs RHE]
275°C
Post
e)
i [mA cm$_{elec}^{-2}$]
E [V vs RHE]
PtNi
Post
f)
i [mA cm$_{elec}^{-2}$]
E [V vs RHE]
PtNi
Post

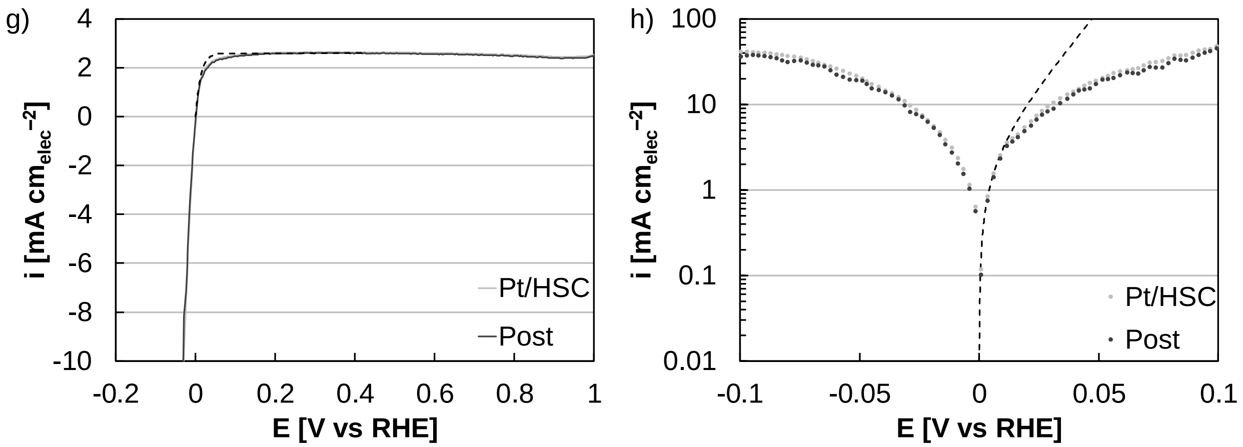

Figure S17. Linear sweep voltammograms and Butler-Volmer plots of (a–b) acid leached Pt-Ni nanowires (Acid), (c–d) hydrogen annealed Pt-Ni nanowires (275°C), (e–f) as-synthesized Pt-Ni nanowires (PtNi), and (g–h) carbon-supported Pt (Pt/HSC), prior to and following durability testing (Post). Durability testing consisted of 30,000 cycles in the potential range -0.2–0.2 V vs RHE in RDE half-cells.

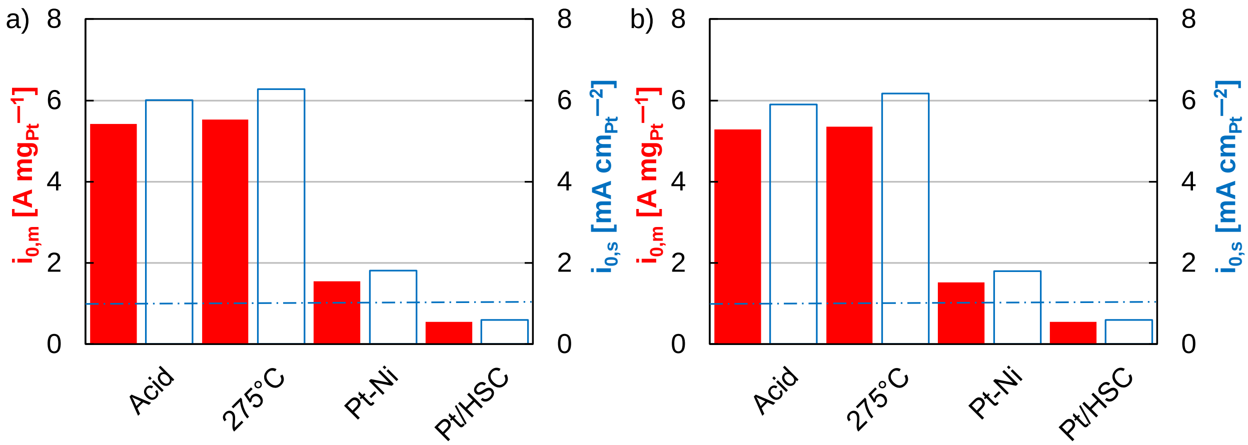

Figure S18. HER-HOR mass (red) and site-specific (blue) exchange current densities of acid leached Pt-Ni nanowires (Acid), hydrogen annealed Pt-Ni nanowires (275°C), as-synthesized Pt-Ni nanowires (Pt-Ni), and carbon-supported Pt (Pt/HSC), (a) prior to and (b) following durability testing. Durability testing consisted of 30,000 cycles in the potential range -0.2–0.2 V vs RHE in RDE half-cells.

Table S3. Equivalent circuit model fits of impedance data from MEAs with acid leached Pt-Ni nanowires, Pt/HSC, and Ni nanoparticle -based cathodes. MEAs were tested in electrolysis mode with Co anodes (0.4 mg_{Co} cm^{-2}), NREL Gen 2 PFAEMs and ionomers (ionomer to catalyst ratio of 0.22), Toray transport layers, and Ni flow fields. Cathode catalyst layers were sprayed to loadings of 0.1 (Pt-Ni, Pt/HSC) and 0.2 (Ni) mg_M cm^{-2}.

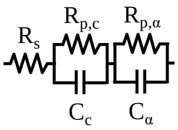

	R_s [Ω cm^2]	$R_{p,c}$ [Ω cm^2]	C_c [mF cm^{-2}]	$R_{p,\alpha}$ [Ω cm^2]	C_α [mF cm^{-2}]
Pt-Ni (Acid)	0.135	0.013	2.2	0.972	1.5
Pt/HSC	0.135	0.166	0.5	0.968	3.5
Ni	0.135	0.561	0.1	0.975	0.6

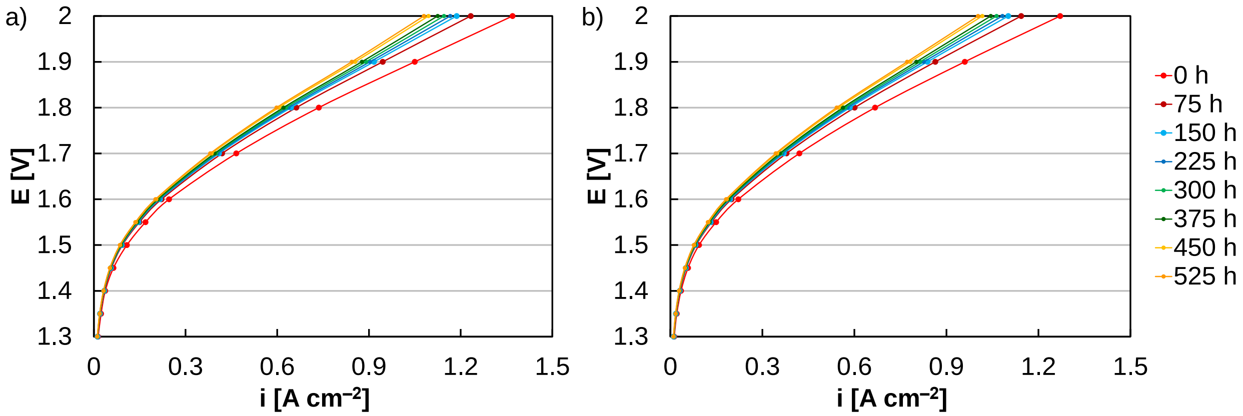

Figure S19. Polarization curves of MEAs with (a) acid leached Pt-Ni nanowires and (b) Pt/HSC -based cathodes during extended operation (2 V hold). MEAs were tested in electrolysis mode with Co anodes (0.4 mg_{Co} cm^{-2}), NREL Gen 2 PFAEMs and ionomers (ionomer to catalyst ratio of 0.22), Toray transport layers, and Ni flow fields. Cathode catalyst layers were sprayed to loadings of 0.1 (Pt-Ni, Pt/HSC) and 0.2 (Ni) mg_M cm^{-2}.
